# Restoration of RNA helicase DDX5 suppresses hepatitis B virus (HBV) biosynthesis and Wnt signaling in HBV-related hepatocellular carcinoma

**DOI:** 10.7150/thno.49629

**Published:** 2020-09-01

**Authors:** Saravana Kumar Kailasam Mani, Bingyu Yan, Zhibin Cui, Jiazeng Sun, Sagar Utturkar, Adrien Foca, Nadim Fares, David Durantel, Nadia Lanman, Philippe Merle, Majid Kazemian, Ourania Andrisani

**Affiliations:** 1 Department of Basic Medical Sciences.; 2 Department of Biochemistry.; 3 Department of Computer Science.; 4 Purdue Center for Cancer Research, Purdue University, West Lafayette, IN 47907.; 5 Cancer Research Center of Lyon UMR Inserm 1052 - CNRS 5286.; 6 Department of Hepatology, Hôpital de la Croix-Rousse, Hospices Civils de Lyon, Université Lyon 1, Lyon, France

**Keywords:** Hepatitis B virus, Hepatocellular Carcinoma, RNA helicase DDX5, miR17~92/miR106b~25 & antagomirs, Wnt/β-catenin signaling.

## Abstract

**Rationale:** RNA helicase DDX5 is downregulated during hepatitis B virus (HBV) replication, and poor prognosis HBV-related hepatocellular carcinoma (HCC). The aim of this study is to determine the mechanism and significance of DDX5 downregulation for HBV-driven HCC, and identify biologics to prevent DDX5 downregulation.

**Methods:** Molecular approaches including immunoblotting, qRT-PCR, luciferase transfections, hepatosphere assays, Assay for Transposase-Accessible Chromatin sequencing (ATAC-seq), and RNA-seq were used with cellular models of HBV replication, HBV infection, and HBV-related liver tumors, as well as bioinformatic analyses of liver cancer cells from two independent cohorts.

**Results:** We demonstrate that HBV infection induces expression of the proto-oncogenic miR17~92 and miR106b~25 clusters which target the downregulation of DDX5. Increased expression of these miRNAs is also detected in HBV-driven HCCs exhibiting reduced *DDX5* mRNA. Stable DDX5 knockdown (DDX5^KD^) in HBV replicating hepatocytes increased viral replication, and resulted in hepatosphere formation, drug resistance, Wnt activation, and pluripotency gene expression. ATAC-seq of DDX5^KD^ compared to DDX5 wild-type (WT) cells identified accessible chromatin regions enriched in regulation of Wnt signaling genes. RNA-seq analysis comparing WT versus DDX5^KD^ cells identified enhanced expression of multiple genes involved in Wnt pathway. Additionally, expression of *Disheveled*, *DVL1*, a key regulator of Wnt pathway activation, was significantly higher in liver cancer cells with low *DDX5* expression, from two independent cohorts. Importantly, inhibitors (antagomirs) to miR17~92 and miR106b~25 restored DDX5 levels, reduced *DVL1* expression, and suppressed both Wnt activation and viral replication.

**Conclusion***:* DDX5 is a negative regulator of Wnt signaling and hepatocyte reprogramming in HCCs. Restoration of DDX5 levels by miR17~92 / miR106b~25 antagomirs in HBV-infected patients can be explored as both antitumor and antiviral strategy.

## Introduction

Hepatocellular carcinoma (HCC) is a leading type of primary cancer with increasing incidence globally 1. Chronic Hepatitis B virus (HBV) infection remains one of the major etiologic factors in HCC pathogenesis 2. Despite the HBV vaccine, the WHO estimates that globally 250 million people are chronically infected with HBV. Curative treatments for early stage HCC include liver resection, transplantation, or local ablation. However, high recurrence rates after resection compromise patient outcomes. In advanced stage HCC, multi-kinase inhibitors including sorafenib 3, regorafenib 4, cabozantinib 5, lenvatinib or anti-angiogenic monoclonal antibodies such as ramucirumab 6 offer only palliative benefits. Thus, a compelling need exists to determine key molecular drivers of HCC pathogenesis, in order to design effective, targeted therapies that suppress both virus biosynthesis and liver cancer pathogenesis.

A cellular mechanism hijacked by HBV that regulates both host and viral gene transcription 7 involves the chromatin modifying Polycomb Repressive Complex 2 (PRC2) complex 8 and RNA helicase DDX5 9. PRC2 represses transcription of genes by trimethylation of histone H3 on lysine 27 (H3K27me3). During HBV infection, viral covalently closed circular DNA (cccDNA) serving as template for viral transcription, assumes chromatin-like structure 10. Histone modifications associated with the HBV cccDNA/minichromosome determine viral transcription and replication rate 11. Our previous studies have shown that HBV replicating cells and HBV-related HCCs exhibit reduced PRC2 activity, resulting in de-repression of host PRC2 target genes 12-14. Moreover, knockdown of the essential PRC2 subunit SUZ12 enhances HBV replication 12, 13, suggesting loss of PRC2-mediated gene repression is advantageous for viral growth. Loss of PRC2 function occurs by proteasomal degradation of SUZ12, dependent on cellular polo-like-kinase1 (PLK1) 15, a host pro-viral factor 16. RNA helicases regulate a wide range of pathways 17. For example, DDX5 interacts with SUZ12, contributing to enhanced SUZ12 protein stability 7. Reduced DDX5 and SUZ12 protein levels correlate with enhanced viral transcription and replication, while in clinical samples, reduced *DDX5* expression correlates with hepatocyte de-differentiation, expression of PRC2 target genes including *EpCAM,* a hepatic Cancer Stem Cell (hCSC) marker 18, and poor patient prognosis 7. These observations suggest a role for DDX5 both in HBV replication and HBV-induced HCC.

In this study, we investigated how HBV infection mediates DDX5 downregulation, and the consequences of DDX5 downregulation for the infected hepatocyte. We show that HBV replication induces the expression of proto-oncogenic miR-17~92 and its paralog miR106b~25 19 which directly target the three prime untranslated region (3'-UTR) of *DDX5*. miRNAs silence gene expression post-transcriptionally, regulating an array of biological processes, and linked to various diseased states including cancer 20. miR17~92 is induced by the proto-oncogene c-Myc 21, and miR106b~25 is encoded within intron 13 of minichromosome maintenance complex component 7 (*MCM7*) 22. Importantly, both miRNAs are upregulated in HBV-induced HCCs 19, 23, and their over-expression is associated with liver fibrosis, cirrhosis, and HCC 19, 24. Biogenesis of mature miRNAs involves processing of primary miRNA (pri-miRNA) by the microprocessor complex 20. DDX5 is a critical component of this complex, and importantly, genes involved in the microprocessor complex are haploinsufficient tumor suppressors 25, 26. Herein, we show DDX5 downregulation imparts cancer stem cell properties to hepatocytes, including hepatosphere formation, resistance to sorafenib and cisplatin, expression of pluripotency genes, and activation of Wnt signaling. Antagomirs (inhibitors) to these miRNAs restore DDX5 levels in HBV replicating cells, suppressing Wnt pathway activation and virus biosynthesis, i.e., acting as both antitumor and antiviral agents.

## Methods

### Cell lines

Human HCC cell lines HepAD38 27, HepG2 7, HepG2-NTCP clone 7 28, Huh7, and HepaRG cells 29 were grown as described. Cell lines were routinely tested for mycoplasma. HepAD38 cell line and its derivatives were authenticated by short tandem repeat (STR) analysis performed by ATCC.

### Cell transfection and infection assays

HepG2 and HepAD38 cells (5x10^4^ cells) were co-transfected with Renilla luciferase (25 ng), Luc-3'UTR-*DDX5* (25 ng), and control (Ctrl) vectors or plasmid encoding miR106b~25 or miR17~92, using Lipofectamine 3000 (Life Technologies). In HepAD38 cells 27, HBV replication was induced by tetracycline removal 48 h prior to transfection. Luciferase activity was measured 48 h after transfection using Dual Luciferase Assay system as per manufacturer's protocol (Promega), and normalized to Renilla luciferase. Plasmids used are listed in Supporting **Table S1**. Infection assays of HepaRG and HepG2-NTCP cell lines were performed as described 28, 29, employing 100 HBV genome equivalents per cell.

### Wnt reporter assay

HBV replicating HepAD38 cells (5x10^4^ cells, day 3 of HBV replication) were co-transfected with TOPflash vector (25 ng) containing TCF-binding sites upstream of firefly luciferase, and Renilla luciferase vector (25 ng). Ctrl siRNA (40 nM) or DDX5 siRNA (40 nM) were co-transfected with Renilla and Firefly luciferase vectors using RNAiMax (Life Technologies). Luciferase activity was measured 48 h after transfection using Dual Luciferase Assay system as per manufacturer's protocol (Promega), and normalized to Renilla luciferase. Plasmids used are listed in **Table S1**.

### Sphere assay

HBV replicating HepAD38 cells (1x10^3^) were seeded in ultra-low attachment 6-well plates (Corning). Cisplatin (10 μM) and Sorafenib (2.5 μM) were replaced every 3 days for 2 weeks, using sphere media containing DMEM/F12 (90% v/v), Penicillin/Streptomycin (1% v/v), G418 50 mg/mL (0.8% v/v), Fibroblast Growth factor 100 ng/μL (0.02% v/v), B27 (1X), and Epidermal growth factor 100 ng/μL (0.02% v/v).

### Cell viability assay

HBV replicating HepAD38 cells (1x10^4^) seeded in 96-well plates were treated with cisplatin (40 μM), sorafenib (7.5 μM), or DMSO for 24 h (day 5 of HBV replication). Growth inhibition was measured at 490 nm by CellTiter 96 AQueous One Solution Cell Proliferation assay (Promega). 100% viability refers to A_490_ value of DMSO-treated cells. Background absorbance was measured from wells containing media and MTS without cells.

### Immunoblot analysis and Immunofluorescence microscopy

Methods are described in detail in Supplementary Material section. Antibodies employed are listed in **Table S2**.

### RNA extraction and qRT-PCR

Detailed methods are described in Supplementary Material section; primer sequences are listed in **Table S3**, and reagents, chemical inhibitors and kits in **Table S4**.

### RNA-seq analysis

HepAD38 cells, wild type (WT) and DDX5 knockdown (KD5) grown +/- tetracycline for 10 days to induce HBV replication 27. Sorafenib (2.5 μM) treatment was for three days prior to harvesting. Three independent biological replicates were prepared for RNA isolation and RNA sequencing. Total RNA submitted to Purdue Genomics Core Facility for quality assessment and next-generation sequencing. Paired-end 2x50 bp sequencing performed using a HiSeq2500 system (Illumina). Data quality control performed using FastQC v0.11.8. The RNA expression level in each library estimated by “rsem-calculate-expression” procedure in RSEM v1.3.112 using default parameters except “--bowtie-n 1 -bowtie-m 100 -seed-length 28 --paired-end”. The bowtie index required by RSEM software generated by “rsem-prepare-reference” on all RefSeq genes, obtained from UCSC table browser on April 2017. EdgeR v3.24.013 package used to normalize gene expression among all libraries and identify differentially expressed genes among samples with following constraints: fold change > 1.5, FDR < 0.05 and TPM > 1. Gene set enrichment analysis (GSEA) performed using GSEA software 30.

### ATAC-seq analysis

Two independent biological replicates of WT HepAD38 and DDX5 knockdown (KD5) cells used for ATAC-seq analyses. Protocol and method of ATAC-seq data analysis described in Supplementary Material section.

### Data availability

All sequencing data are available through the NCBI Gene Expression Omnibus (GEO) database (accession number GSE131257).

### Chromatin immunoprecipitation (ChIP)

ChIP assays were performed using Millipore ChIP Assay Kit (catalog no.: 17-295). Antibodies used listed in **Table S2**. ChIP primers used are described in 21.

### Statistical analysis

**Figure 1:**
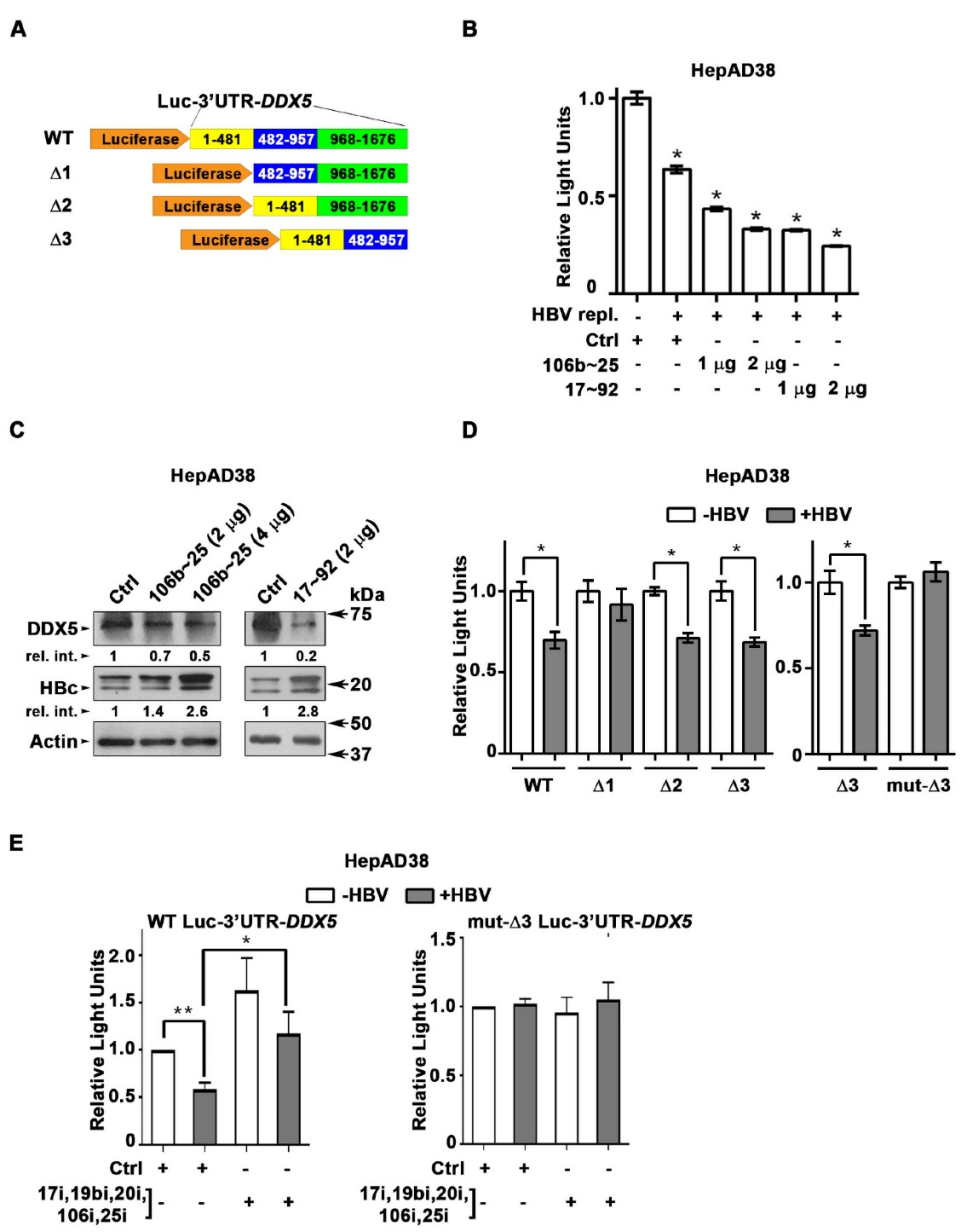
***DDX5* is target of miR106b~25 and miR17~92 in HBV replicating cells. (A)** Luc-3'UTR-*DDX5* containing the WT 3'UTR, indicated deletions Δ1, Δ2, Δ3, and site directed changes of nucleotides 129-135 (mut-Δ3). **(B)** Transient transfections of Luc-3'UTR-DDX5 co-transfected with Renilla luciferase, and plasmids expressing miR106b~25 or miR-17~92 in HepAD38 cells without (-) and with (+) HBV replication by tetracycline removal for 2 days.** (C)** Immunoblots of DDX5 and HBc following transfection of plasmids expressing miR106b~25 or miR-17~92, in HepAD38 cells with HBV replication for 2 days. Relative intensity (rel. int.) quantified vs. actin using ImageJ software. **(D)** Luc-3'UTR-*DDX5* containing the WT 3'UTR, indicated deletions Δ1, Δ2, Δ3, and site directed changes of nucleotides 129-135 (mut-Δ3), co-transfected with Renilla luciferase in HepAD38 cells with (+) or without (-) HBV replication for 2 days (n=3). * *P <* 0.05; Error bars indicate Mean ± SEM. **(E)** WT and mut-Δ3 Luc-3'UTR-*DDX5* co-transfected with Renilla luciferase and 10 nM each of indicated miRNA inhibitors (antogomirs) or 50 nM control inhibitor (Ctrli) in HepAD38 cells with (+) or without (-) HBV replication for 2 days (n=3). * *P <* 0.05; Error bars indicate Mean ± SEM.

**Figure 2:**
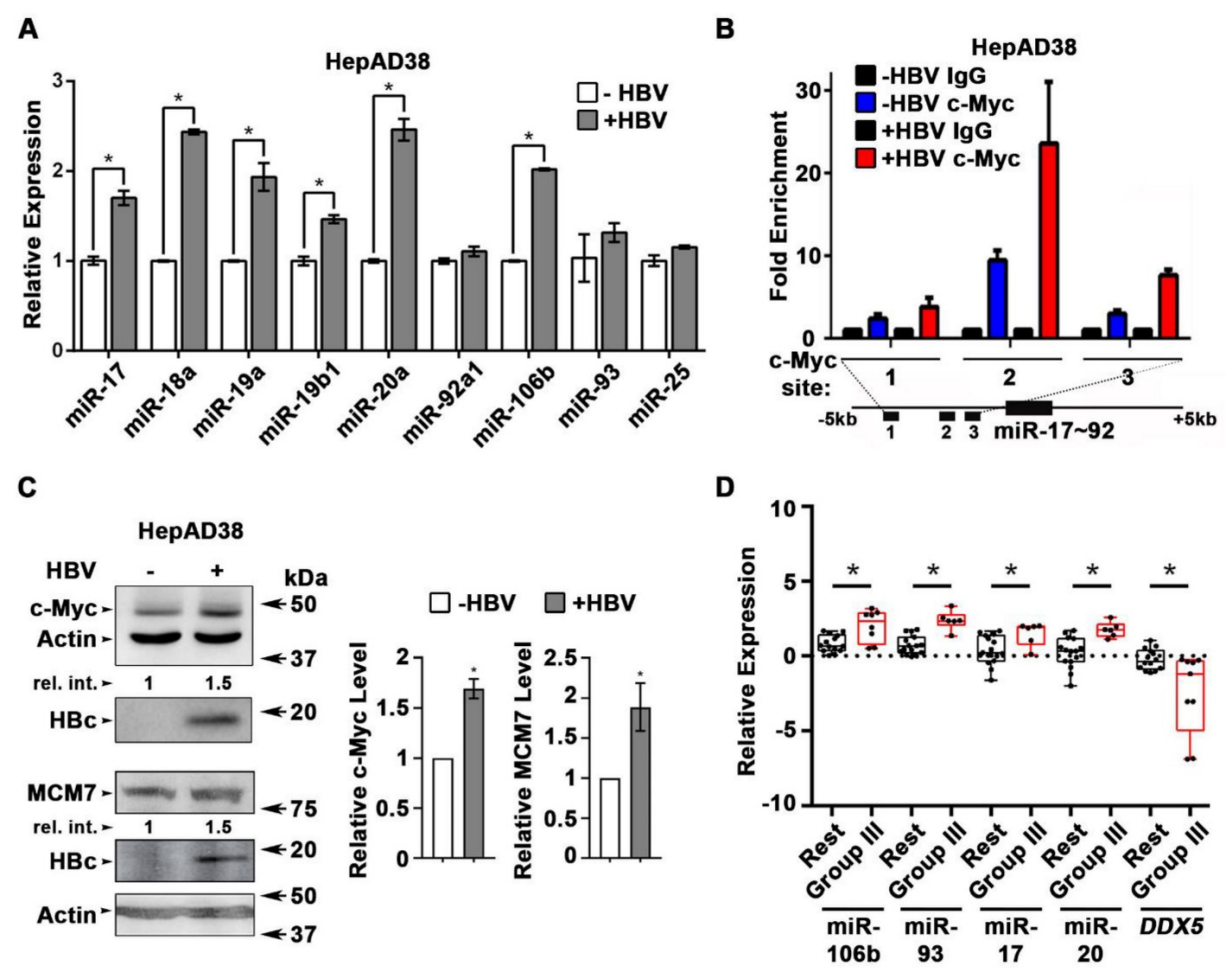
** HBV replication induces expression of miR106b~25 and miR17~92. (A)** qRT-PCR of indicated miRNAs in HepAD38 cells without (-) and with (+) HBV replication for 5 days (n=3). **(B)** ChIP assays in HepAD38 cells with (+) or without (-) HBV replication for 5 days, using c-Myc antibody and primer sets 1-3 spanning c-Myc binding sites 21. IgG was negative control. (n=3). Schematic representation of genomic interval encompassing the miR17~92 cluster. RT-PCR amplicons represented by numbered lines. **(C)** Immunoblots of c-Myc and MCM7 using lysates from HepAD38 cells with (+) or without (-) HBV replication for 7days. Quantification by ImageJ software of three independent experiments. **(D)** qRT-PCR of indicated miRNAs, and *DDX5* in HBV-related HCC tumor vs. peritumor samples. Tumor samples were grouped according to Mani et al, 14 into Group III vs. Rest (refer also to **Figure S2C**). * *P <* 0.05; Error bars indicate Mean ± SEM.

**Figure 3:**
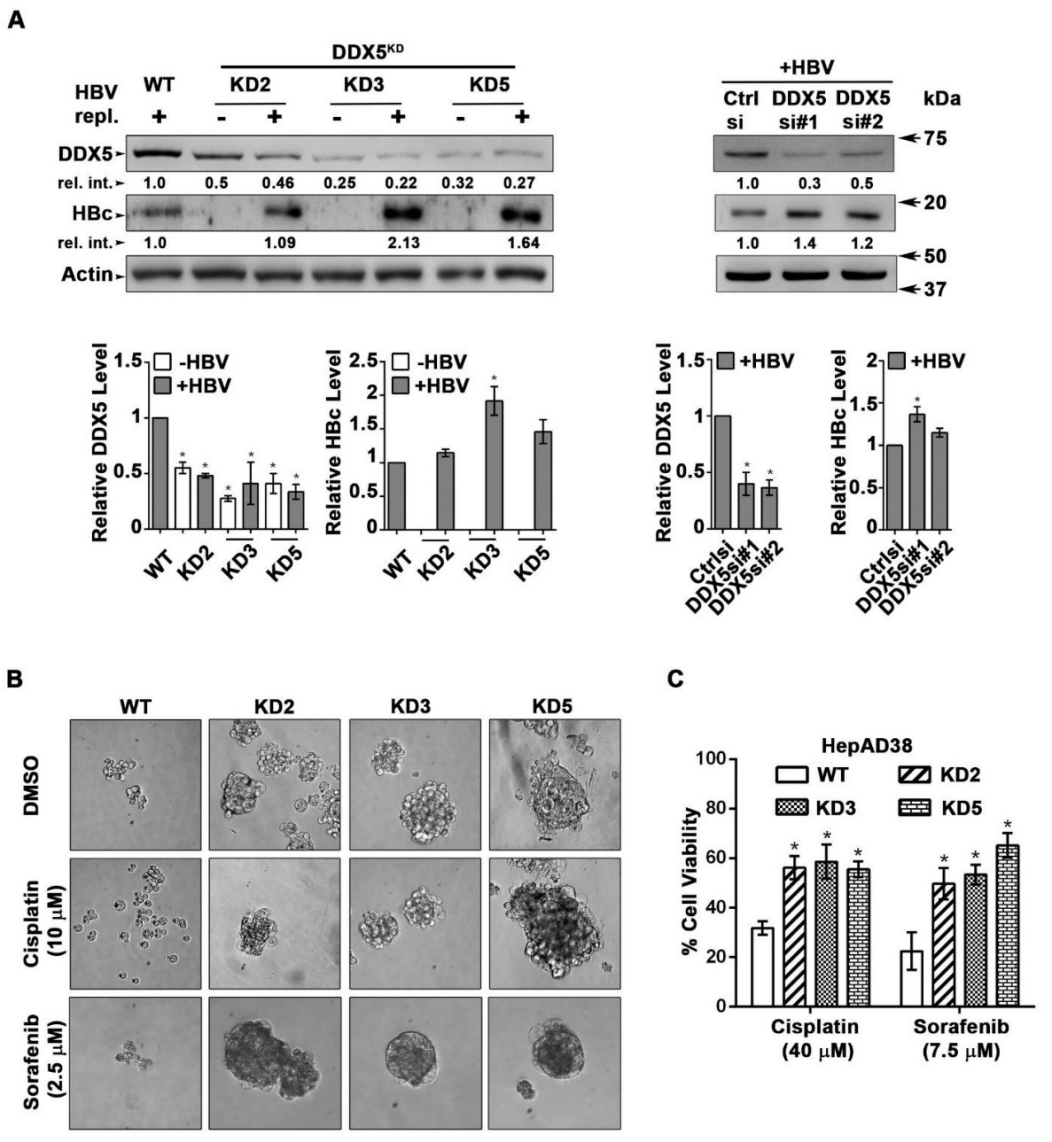
**DDX5 knockdown confers stem cell-like properties. (A)** Immunoblots of DDX5 and HBc in WT and DDX5 knockdown (KD2, KD3 and KD5) HepAD38 cells, and in WT HepAD38 cells following transient transfection of two different siRNAs for DDX5 or control siRNA (Ctrl), without (-) and with (+) HBV replication for 5 days. Panels shown below the immunoblots are cumulative quantification of three independent biological replicates.** (B)** HBV replicating WT, KD2, KD3 and KD5 HepAD38 cells grown for 14 days in hepatic sphere media using ultra-low attachment plates, with cisplatin (10 μM) or sorafenib (2.5 μM). Shown is a representative assay of three independent biological replicates.** (C)** Proliferation (MTS) assays performed with WT, KD2, KD3 and KD5 HepAD38 cells grown with (+) HBV replication for 5 days. Cells were treated with cisplatin (40 μM), sorafenib (7.5 μM) or DMSO for 24 h (n=3). *P < 0.05; Error bars indicate Mean ± SEM.

One-way ANOVA with Sidak's multiple comparison test with single pooled variance was performed using GraphPad Prism version 5.0, comparing mean of each sample to mean of control **(Figure 1B, 1D)**. Two-way ANOVA with Sidaks's multiple comparison test was performed comparing: (i) mean of each microRNA in infected to uninfected samples **(Figure 2A)** and** (Figure S2A)**, (ii) mean of each cell line to mean of WT cells** (Figure 3C), (Figure 5A),** and** (Figure 5C)**, (iii) mean of each siRNA to control siRNA** (Figure 5A and B)**, and (iv) mean of each inhibitor to mean of control inhibitor **(Figure 8C and D)**. Two tailed t-test with Welch's correction was used to determine significance in **Figure 2D.** Results were considered statistically significant if p < 0.05.

## Results

### DDX5 downregulation in HBV replicating cells by miR106b~25 and miR17~92

DDX5 is downregulated during HBV replication, and reduced levels of DDX5 in HBV-related HCCs tended towards poor patient prognosis 7. Employing the target prediction algorithm TargetScan, a highly conserved seed sequence for miR17~92 and its paralog miR106b~25 19, 31 was identified in 3' UTR of *DDX5,* located at nucleotides 129-135 (**Figure S1A**). To test whether miR106b~25 and miR17~92 downregulate DDX5, expression vectors encoding each of these miRNAs were co-transfected with expression vectors encoding the Firefly luciferase gene fused to 3'UTR of *DDX5* (Luc-3'UTR-*DDX5*), and a Renilla luciferase vector for normalization of luciferase activity **(Figure 1A)**. Overexpression of either miR106b~25 or miR17~92 in HepG2 cells reduced Firefly luciferase activity, indicating a functional seed sequence in 3'UTR of *DDX5* **(Figure S1B)**. Furthermore, transfection of Luc-3'UTR-*DDX5* vector in HepAD38 cells exhibited reduced luciferase activity upon induction of HBV replication, consistent with upregulation of both miRNAs in HBV-induced HCCs 19, 23 and their role in targeting DDX5. Overexpression of miR106b~25 or miR17~92 clusters further reduced luciferase activity** (Figure 1B)**. Importantly, overexpression of these miRNAs in HBV replicating HepAD38 cells 27 reduced protein level of endogenous DDX5, while levels of viral core antigen (HBc) were increased **(Figure 1C)**, suggesting loss of DDX5 is advantageous to viral biosynthesis. Deletion analyses and site directed mutagenesis of 3'UTR of *DDX5* confirmed the presence of miRNA seed sequence at nucleotides 129-135 (mut-Δ3), analyzed in the context of HBV replication (**Figure 1D**). Inhibitors (antagomirs) for miR106b~25 and miR17~92 transfected in HBV replicating cells reversed the reduction in luciferase activity from the WT Luc-3'UTR*-DDX5*, without an effect on the mut-Δ3 construct lacking the conserved seed sequence **(Figure 1E)**. Together, these results indicate that HBV replication downregulates *DDX5* mRNA via induction of miR106b~25 and miR17~92.

### HBV replication induces expression of miR106b~25 and miR17~92

Next, we quantified the expression of individual members of miR17~92 and miR106b~25 clusters in HBV replicating HepAD38 cells** (Figure 2A)**, and HBV infected HepaRG cells **(Figure S2A)**. Five out of six members of miR17~92 cluster and one out of three members of miR106b~25 cluster were significantly induced in HBV replicating HepAD38 cells **(Figure 2A)**, while two members of miR17~92 were induced more than two-fold in HBV infected HepaRG cells **(Figure S2A)**. To understand how HBV infection increased miR17~92 expression, we focused on the miR17~92 promoter region which contains three binding sites for c-Myc, often overexpressed in liver cancer and a key regulator of miR17~92 21. Two out of these three sites (i.e. sites #2 and #3) exhibited further increased c-Myc occupancy in HBV replicating HepAD38 cells **(Figure 2B)**, consistent with induction of miR17~92 members **(Figure 2A)**, and the increased c-Myc expression detected by immunoblots **(Figure 2C)**. miR106b~25 is encoded by intron13 of the *MCM7* gene and when* MCM7* is overexpressed, it also increases expression of miR106b~25 22. To determine whether *MCM7* is overexpressed, we quantified MCM7 protein levels in lysates from HBV replicating HepAD38 cells** (Figure 2C)**. Approximately 2-fold increased MCM7 protein level was observed, in agreement with the well-established activation of cellular mitogenic pathways by the viral HBx protein 32, 33, supporting the increased expression of miR106b~25 in HBV replicating cells** (Figure 2A)**. In addition to DDX5 **(Figure 1)**, the increased expression of these miRNAs also downregulates other known cellular targets, including PTEN 22 and LKB1 34. Indeed, HBV replicating cells display reduced PTEN and LKB1 levels, which regulate AKT phosphorylation **(Figure S2B)**, and AMPK activity 35, respectively. Importantly both of these pathways are known to exert a positive effect on HBV replication 36-38. Overall, these data indicate that HBV replication induces expression of miR106b~25 and miR17~92.

To determine whether miRNA-mediated downregulation of DDX5 is clinically relevant, the level of these miRNAs was quantified in HBV-related HCCs. Our previous study identified a hepatic cancer stem cell (hCSC)-like gene signature to be associated with tumor recurrence after surgery in HBV-related HCCs 14. In that study (14), we denoted liver tumors expressing the hCSC-like gene signature as “Group III”, while tumors that did not express the hCSC-like signature 14 were referred to as the “Rest” **(Figure S2C)**. Herein, we quantified miRNA expression in the same HBV-related HCCs. Significantly, “Group III” HBV-related HCCs, in comparison to the “Rest”, exhibited increased expression of miR106b~25 and miR17~92 and reduced levels of *DDX5* mRNA **(Figure 2D).** Considering that miRNAs act by reducing either mRNA stability or translation of their targets, these clinical data strongly suggest an inverse correlation between high expression of miR106b~25 and miR17~92 vs. reduced *DDX5* levels **(Figure S2D)**, and, in turn, tumor aggressiveness.

### DDX5 knockdown induces viral biosynthesis and confers cancer stem cell-like properties to hepatocytes

We next studied the functional significance of DDX5 loss to viral biosynthesis, and hepatosphere formation. We derived three stable DDX5 knockdown cell lines (DDX5^KD^) from HepAD38 cells, referred to as KD2, KD3 and KD5** (Figure 3A)** using three different shRNAs. To assess whether DDX5 downregulation affected viral replication, we quantified HBc levels by immunoblots. HBc levels were increased after DDX5 knockdown, both in the stable cell lines **(Figure 3A)** and after transient transfection of DDX5 siRNAs #1 and #2 **(Figure 3A)**. These results also agree with the increased HBc levels observed upon overexpression of miR106b~25 and miR17~92 in HBV replicating HepAD38 cells **(Figure 1C)**. Collectively, these data suggest that DDX5 acts as a host restriction factor for HBV biosynthesis.

Since DDX5 was shown to act as a roadblock to pluripotency 39, we examined whether loss of DDX5 promotes a stem cell-like phenotype in HBV replicating hepatocytes. WT HepAD38 cells failed to form hepatospheres in ultra-low attachment plates. By contrast, DDX5^KD^ cells (KD2, KD3, and KD5) formed robust hepatospheres that survived treatment with chemotherapeutic drugs cisplatin (10 μM) and sorafenib (2.5 μM) **(Figure 3B)**. Furthermore, proliferation assays demonstrated reduced sensitivity to cisplatin and sorafenib upon DDX5 downregulation **(Figure 3C and Figure S3)**, a characteristic feature of cancer stem cells (CSCs).

**Figure 4:**
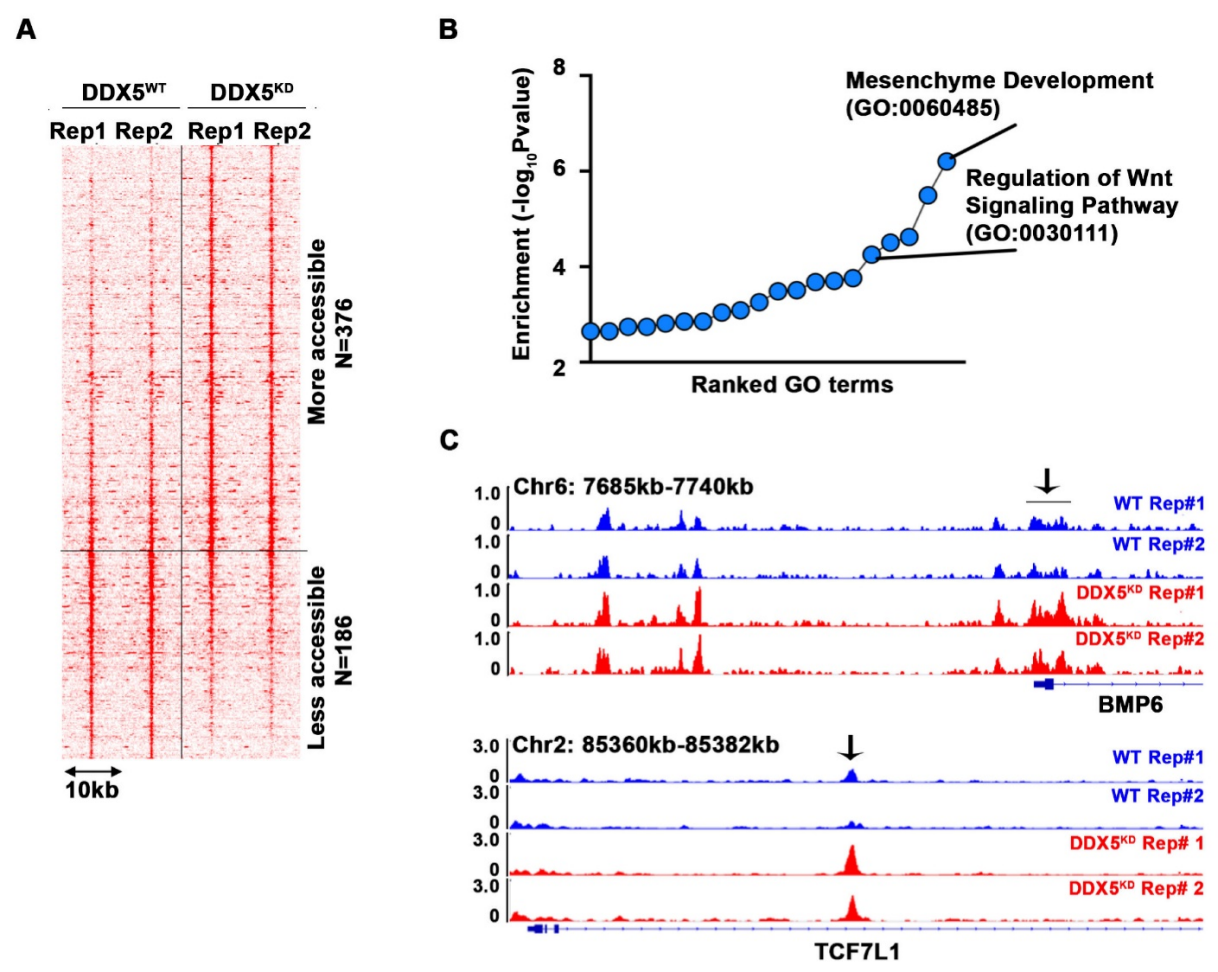
** DDX5 downregulation alters chromatin accessibility near genes associated with Wnt pathway. (A)** Heatmap showing signal intensity of each differential ATAC peak (n=2 biological replicates) and clustering of peaks into two groups, more accessible group (top) and less accessible group (bottom) in DDX5^KD^ cells.** (B)** Pathway analysis of genes neighboring the top group with representative examples of open chromatin regions (OCRs) **(C)** Highlighted with arrow are OCRs corresponding to ATAC peaks in heatmap.

To determine pathways contributing to the observed stemness characteristics, we performed the Assay for Transposase-Accessible Chromatin sequencing (ATAC-seq) and compared the chromatin accessibility of WT vs. DDX5^KD^ cells **(Figure 4A and Table S5)**. Interestingly, 376 loci became more accessible in DDX5^KD^ cells, while 186 loci became less accessible. Next, we performed pathway analysis of the genes neighboring these accessible regions. We found that regulation of Wnt signaling pathway was the fifth-most enriched pathway among all biological pathways assessed **(Figure 4B)**. Examples of Wnt pathway genes exhibiting changes in chromatin accessibility are shown in **Figure 4C**, suggesting DDX5 may regulate Wnt signaling by affecting chromatin accessibility of genes involved in Wnt signaling.

### DDX5 downregulation activates Wnt signaling in liver cancer cell lines

**Figure 5:**
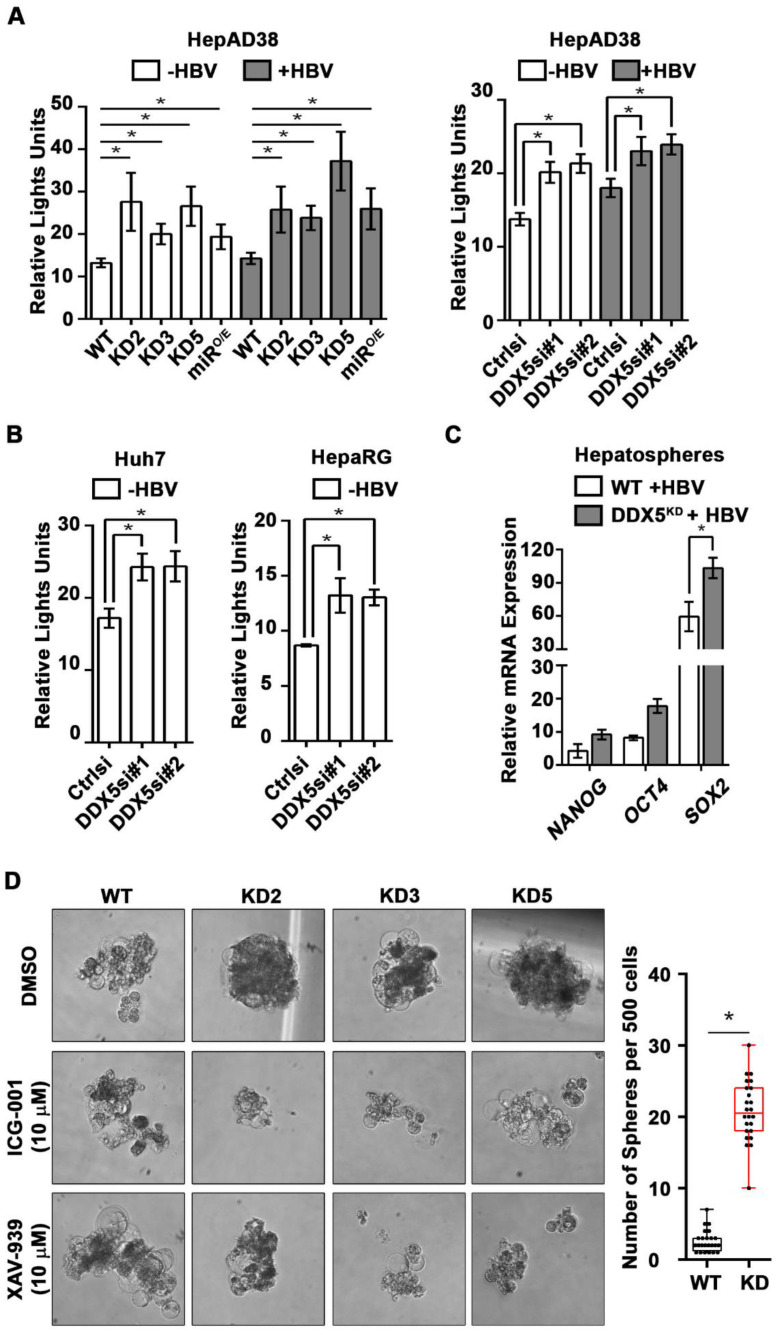
** Downregulation of DDX5 activates Wnt signaling. (A-B)** Transient co-transfections of TOPFlash and Renilla luciferase reporters in HepAD38 cells (WT, KD2, KD3, KD5 and miR^O/E^)** (A),** and Huh7 and HepaRG cells** (B)**, transfected with *DDX5si#1, DDX5si#2* or negative control siRNA (Ctrlsi). Luciferase activity from three independent assays, measured on day 5 of HBV replication.** (C)** qRT-PCR of *OCT4 NANOG, SOX2* expression in WT and DDX5^KD^ HepAD38 hepatospheres with (+) HBV replication for 14 days. **(D)** WT, KD2, KD3 and KD5 HepAD38 cells grown for 14 days in hepatic sphere media using ultra-low attachment plates, in presence of Wnt inhibitors ICG-001 (10 μM) or XAV-939 (10 μM). Shown is a representative assay (left) and quantification of DDX5^KD^ hepatospheres (right) from three independent biological replicates. *P < 0.05; Error bars indicate Mean ± SEM.

Based on the ATAC-seq results** (Figure 4)**, we examined activation of Wnt/β-catenin signaling, one of the key pathways regulating stemness 40, 41. We performed luciferase assays to assess whether Wnt signaling was upregulated upon reduction of DDX5. Wnt-responsive TOPFlash reporter, containing LEF/TCF binding sites upstream of Firefly luciferase, was co-transfected with control Renilla luciferase into WT HepAD38 cells, HepAD38 cells overexpressing miR106b~25 and miR17~92 **(miR^O/E^, Figure S4A-B)**, and stably or transiently DDX5 knockdown cells. Increased luciferase activity, i.e. increased Wnt/β-catenin pathway activation, was observed upon DDX5 downregulation by stable overexpression of miR106b~25 and miR17~92 (miR^O/E^), stable (KD2, KD3, and KD5) and transient (siRNAs #1 and #2) knockdown of DDX5 **(Figure 5A)**. Furthermore, transient (siRNAs #1 and #2) knockdown of DDX5 in Huh7 and HepaRG cells also increased luciferase expression from the TOPFlash reporter, supporting this mechanism is functional in other liver cancer cell lines** (Figure 5B)**. Interestingly, hepatospheres of HBV replicating DDX5 knockdown cells exhibited higher expression of pluripotency genes, determined by qRT-PCR **(Figure 5C)** and immunoblotting of NANOG, SOX2, OCT4 and hCSC marker CD44 **(Figure S4C)**. Wnt inhibitors, ICG-001 42 and XAV-939 43, targeting different steps of Wnt signaling, suppressed hepatosphere formation, thereby linking Wnt pathway activation to the hCSC phenotype** (Figure 5D, left panel)**. Quantification of hepatosphere formation shows statistically increased hepatosphere formation in DDX5^KD^ cells** (Figure 5D, right panel)**. Taken together, these data indicate that DDX5 maintains hepatocyte differentiation, and DDX5 downregulation promotes hepatocyte deprogramming via activation of Wnt signaling.

### Transcriptomic analyses define dysregulated Wnt signaling in DDX5 knockdown cells

**Figure 6:**
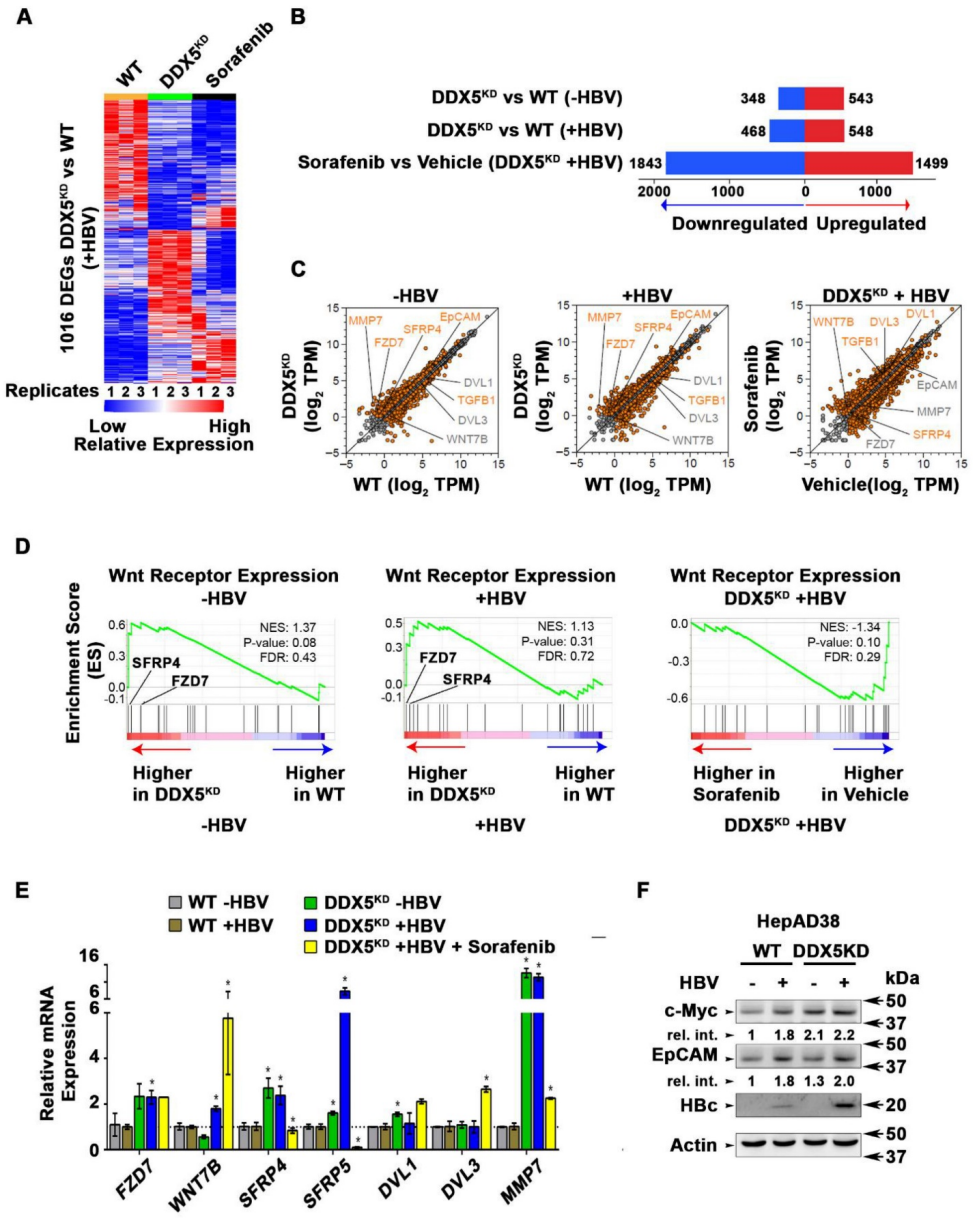
** Transcriptomic analyses define dysregulated Wnt signaling in DDX5 knockdown cells. (A)** Heat map of differentially expressed genes between DDX5^KD^ vs*.* WT HBV replicating cells for 10 days. RNA-seq samples are from three independent biological replicates.** (B)** Differentially expressed genes in three indicated comparisons: DDX5^KD^ vs*.* WT cells in the absence of HBV replication (-HBV), DDX5^KD^ vs*.* WT cells in the presence of HBV replication (+HBV), and DDX5^KD^ HBV replicating cells with or without sorafenib treatment.** (C)** Scatter plot showing mean gene expression values (n=3) in three, above mentioned comparisons. Differentially expressed genes are highlighted in orange (FDR<0.05) and grey (FDR>0.05) **(D)** GSEA for Wnt-activated receptor activity (GO: 0042813), comparing DDX5^KD^ vs*.* WT cells in the absence of HBV replication (left panel), DDX5^KD^ vs*.* WT cells in the presence (+) of HBV replication (middle panel), and DDX5^KD^ HBV replicating cells with or without sorafenib treatment (right panel). **(E)** qRT-PCR validation of indicated genes using RNA isolated from HepAD38 cells, comparing DDX5^KD^ vs*.* WT cells in the absence (-) or presence (+) of HBV replication for 5 days, and with sorafenib (2.5 µM) treatment for 3 days. Expression values calculated for WT -HBV vs. DDX5 -HBV; WT +HBV vs. DDX5 +HBV and DDX5+HBV vs. DDX5+HBV+Sorafenib, using ΔΔCt method. (n=3). **(F)** Immunoblot of indicated proteins in WT and DDX5^KD^ HepAD38 cells, with HBV replication for 5 days. A representative assay from three biologic replicates. Quantification is shown in Fig. S5.

To quantify the global effects of DDX5 knockdown on global gene expression in hepatocytes, we compared the transcriptome of WT vs. DDX5^KD^ HepAD38 cells as a function of HBV replication, and sorafenib treatment using RNA-seq. Three comparisons were performed, namely, DDX5^KD^ vs*.* WT cells in the absence of HBV replication (-HBV), DDX5^KD^ vs*.* WT cells in the presence of HBV replication (+HBV), and DDX5^KD^ HBV replicating cells with or without sorafenib treatment. Nearly 1000 genes were differentially expressed between DDX5^KD^ vs*.* WT cells** (Fold change >1.5; FDR<0.05; Figure 6A-B and Table S6)**. Interestingly, sorafenib treatment exerted a highly pronounced effect on the number of both upregulated and downregulated genes **(Figure 6B)**. Importantly, DDX5 downregulation, irrespective of HBV replication, increased *EpCAM* expression **(Figure 6C)** among other genes, which is a known hepatic cancer stem cell marker 18 and a Wnt/β-catenin signaling target gene 44. To further investigate the effect of DDX5 downregulation on Wnt/β-catenin signaling pathway, we asked whether expression of Wnt receptor genes differed between WT vs. DDX5^KD^ cells, using Gene Set Enrichment Analysis (GSEA) 30. We found that DDX5^KD^ cells have higher expression of several genes involved in Wnt receptor signaling, irrespective of HBV replication, consistent with activation of the Wnt pathway in DDX5^KD^ cells** (Figure 5A and B)**. For example, Wnt receptors (*frizzled*/*FZD*) were among the upregulated genes in DDX5^KD^ cells** (Figure 6D)**, in agreement with earlier studies linking Wnt receptor (*FZD7*) overexpression to Wnt pathway activation in HCCs 45.

The expression of select genes involved in Wnt/β-catenin signaling was validated by qRT-PCR **(Figure 6E)**. Specifically, in DDX5^KD^ cells expression of *FZD7, SFRP4*, *SFRP5, DVL1* and *MMP7* increased irrespective of HBV replication** (Figure 6E)**. Sorafenib treatment of HBV replicating DDX5^KD^ cells increased expression of *WNT7B* ligand, while significantly suppressing expression of *SFRP4* and* SFRP5*** (Figure 6E)**, the negative regulators of Wnt signaling 46. These observations, together with the results of **Figure 5 (A and B)** demonstrate that downregulation of DDX5 is a key event leading to activation of Wnt signaling. To further confirm these findings, we examined by immunoblots protein levels of known Wnt regulated genes, including EpCAM and c-Myc **(Figure 6F and Figure S5)**. We observe increased protein levels of both c-Myc and EpCAM in HBV replicating DDX5^KD^ cells.

### Elevated mRNA expression of *DVL1* in liver cancer cells with low *DDX5* mRNA

**Figure 7:**
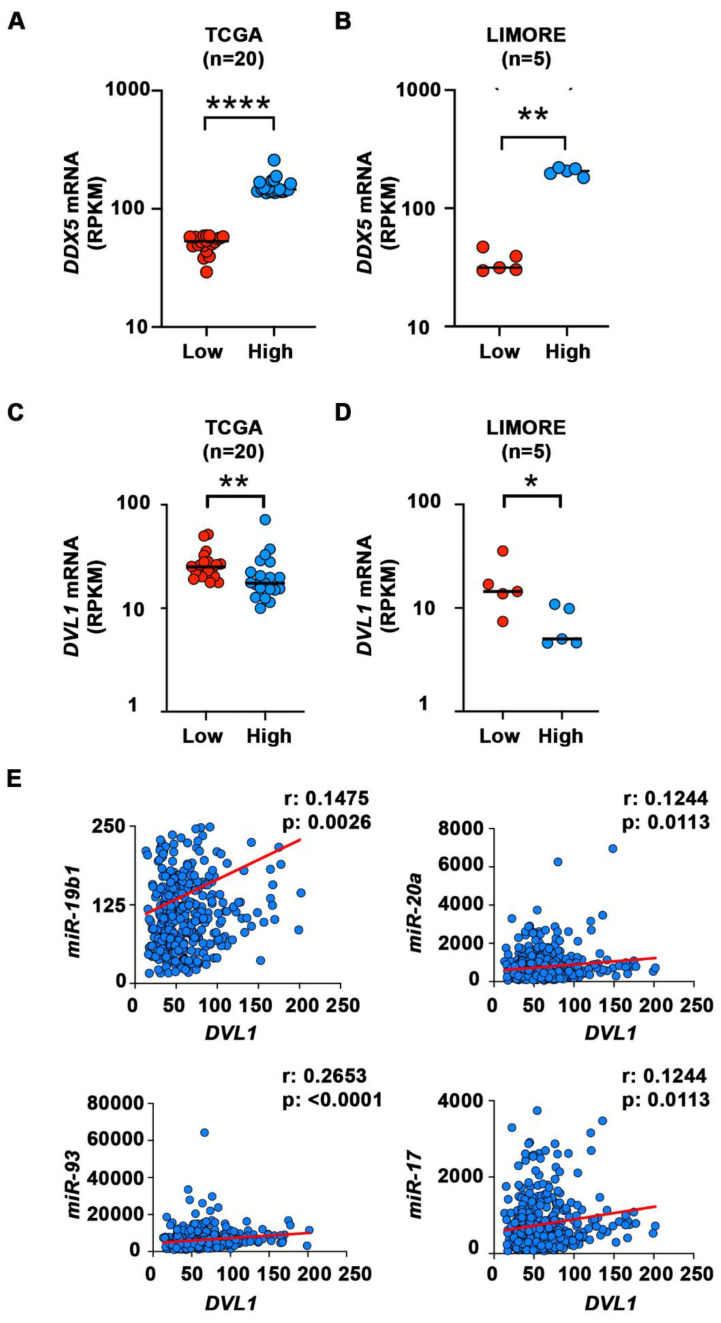
** Elevated mRNA expression of *DVL1* in liver cells with low *DDX5* mRNA. (A-B)** Dot plots showing expression of *DDX5* **(A-B)** and *DVL1* **(C-D)** mRNA in samples with lowest *DDX5* and samples with highest expression of *DDX5* in TCGA (n=20) **(A and C)** and LIMORE** (B and D)** datasets. Median highlighted. *: *P <* 0.05; **: *P <* 0.01; ****: *P <* 0.0001.** (E)** Scatter plots show Spearman correlation between indicated miRNAs and *DVL1* for all liver cancer samples in TCGA.

We next analyzed transcriptomic data of HCCs from The Cancer Genome Atlas (TCGA) 47 and Liver cancer Model Repository (LIMORE) cell line databases 48. We divided the samples into two groups, those with the lowest and those with the highest expression of *DDX5* mRNA **(Figure 7A-B)**. We then compared the expression of several genes from **Figure 6E** that are involved in activation of Wnt signaling in these two groups. Among these genes, *DVL1,* a key positive regulator of Wnt signaling 49-51, was significantly different between the two groups in both TCGA and LIMORE **(Figure 7 C-D)**. Interestingly, samples with the lowest expression of *DDX5* exhibited statistically higher expression of *DVL1*. This result is consistent with the observed increased expression of *DVL1* **(Figure 6E)** and the activation of Wnt signaling** (Figures 4-6)** in the DDX5^KD^ cell lines. To further link *DVL1* expression to the mechanism that mediates DDX5 downregulation, we performed correlation analysis between miR-19b1, miR-93, miR-20a, miR-17 and *DVL1* expression across all HCC samples in TCGA. *DVL1* mRNA expression positively correlates with all four miRNAs** (Figure 7E)**.

### miRNA inhibitors (antagomirs) restore DDX5 in HBV replicating hepatocytes

**Figure 8:**
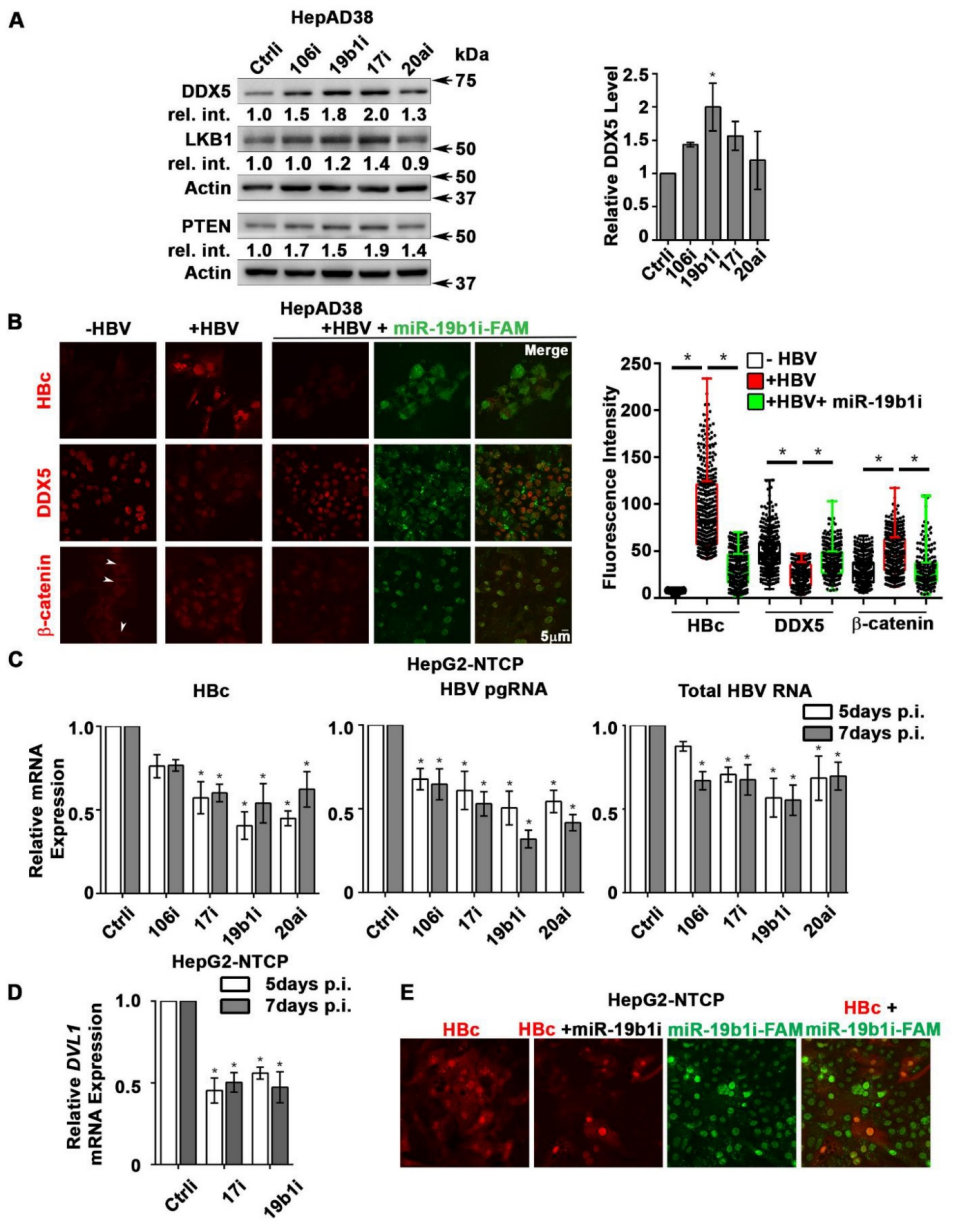
** Antagomirs restore DDX5 in HBV replicating hepatocytes. (A)** Immunoblots of DDX5, LKB1 and PTEN in HepAD38 cells with (+) HBV replication for 5 days. Cells were transfected for 24 h with 50 nM of indicated miRNA inhibitors/antagomirs (106i, 19b1i, 17i, 20ai) or control inhibitor (Ctrli), on day 4 of HBV replication. Cumulative immunoblot quantification of three independent biological replicates for DDX5 shown on right panel, and for LKB1 and PTEN in Figure S6B. *P < 0.05; Error bars indicate Mean ± SEM.** (B)** Immunofluorescence confocal microscopy of HBc, DDX5 and β-catenin in HepAD38 cells without (-) or with (+) HBV replication for 5 days. Fluorescent antagomir for miR-19b1 (miR-19b1i-FAM, 50 nM) transfected on day 4 of HBV replication. White arrows indicate membrane localization of β-catenin. A representative assay from three independent biological replicates. Right panel, quantification of fluorescence intensities from 400 cells (mean gray value per μm^2^), employing ImageJ software. *P < 0.05; Error bars indicate Mean ± SEM.** (C - D)** HepG2-NTCP cells infected with 100 HBV genome equivalents/cell for 5 and 7 days. Infected cells were transfected with antagomirs of indicated miRNAs or Ctrli (50 nM each) 24 h prior to cell harvesting. **(C)** qRT-PCR of HBc RNA, HBV pgRNA, total HBV RNA and **(D)** qRT-PCR of *DVL1* mRNA. Expression values calculated relative to Ctrli at 5 and 7 days p.i. using ΔΔCt method (n=3). *P < 0.05; Error bars indicate Mean ± SEM.** (E)** Immunofluorescence confocal microscopy of HBc in HepG2-NTCP cells at 7 day p.i., infected with 100 HBV genome equivalents per cell. Antagomir miR-19b1i-FAM (50 nM) transfected on day 6 p.i. A representative assay from three independent biological replicates.

DDX5 acts as a host cell restriction factor for HBV replication **(Figures 1C and 3A)**, a barrier to hepatocyte dedifferentiation, and a negative regulator of Wnt signaling **(Figures 4 and 5).** We have also shown that overexpression of miR106b~25 and miR17~92 downregulate DDX5 **(Figure 1)**. However, whether miRNA inhibitors can prevent DDX5 downregulation and associated phenotypes is unclear. To investigate the effect of the miRNA inhibitors (antagomirs), we targeted specific members of miR106b~25 and miR17~92 families. Tumor suppressors LKB1 34 and PTEN 22, well-described targets of miR17~92 and miR106b~25 clusters, respectively, were downregulated in HBV replicating cells **(supporting Figure S6A)**. Transfection of indicated antagomirs restored DDX5 as well as tumor suppressors LKB1 and PTEN in HBV replicating cells **(Figure 8A and Figure S6B)**, suggesting these antagomirs have antitumor potential.

Next, we analyzed the antagomir effect on HBV replication, employing immunofluorescence microscopy of HBc. Co-transfection of combination of antagomirs for miR-106b, miR-17, miR-20a, and miR-19b1 reduced HBc immunostaining by nearly 50% in HepAD38 cells** (Figure S6C)**. Accordingly, we designed antagomirs to miR-19b1 and miR-17 conjugated to a fluorophore (FAM). HepAD38 cells were transfected with miR-19b1i-FAM **(Figure 8B)** or miR-17i-FAM **(Figure S6D)** on day3 of HBV replication. Inhibitor miR-19b1i-FAM reduced HBc immunofluorescence in HBV replicating cells, while the signal for DDX5 increased. Interestingly, miR-19b1i-FAM also reduced nuclear immunostaining of β-catenin in HBV replicating HepAD38 cells **(Figure 8B)**, indicating that restoring DDX5 suppressed Wnt/β-catenin pathway activation. Quantification of the fluorescence intensity demonstrates that the observed effects by miR-19b1i-FAM **(Figure 8B)** and miR-17i-FAM **(Figure S6D)** are statistically significant.

We then utilized the infection model of HepG2-NTCP cells to verify effects of miR106b~25 and miR17~92 antagomirs on virus biosynthesis. HepG2-NTCP cells were infected with 100 HBV genome equivalents per cell; on days 4 and 6 post infection (p.i.), HBV infected cells were transfected for 24 h with antagomirs for miR-106b, miR-17, miR-20a, and miR-19b1 or control inhibitor (Ctrli). Quantification of viral RNA on days 5 and 7 p.i. demonstrated significant reduction in the expression of HBc mRNA, pre-genomic RNA (pgRNA), and total HBV RNA in infected cells transfected with the indicated antagomirs **(Figure 8C)**. Interestingly, antagomir transfected samples also exhibited reduced expression of *DVL1* mRNA (**Figure 8D**), supporting the role of DDX5 as a negative regulator of Wnt signaling. We also monitored HBV biosynthesis in HBV infected HepG2-NTCP cells by fluorescence microscopy for HBc, as a function of transfection of miR-19b1i-FAM** (Figure 8E)** and miR-17i-FAM **(Figure S6E)**. On day 7 p.i., HBV infected cells with strong HBc immunofluorescence (red) exhibited low fluorescence due to miR-19b1i-FAM (green); conversely, cells with strong green fluorescence exhibited weak immunostaining for HBc** (Figure 8D)**. Similar results were obtained with miR-17i-FAM **(Figure S6E)**. Taken together, these results demonstrate that antagomirs for miR106b~25 and miR17~92 restore DDX5 levels in HBV infected cells, attenuating Wnt pathway activation and HBV replication.

## Discussion

In this study, we investigated the mechanism by which HBV infection downregulates the RNA helicase DDX5, and the significance of this downregulation to HCC pathogenesis. RNA helicases are involved in all aspects of RNA metabolism, from transcription, epigenetic regulation, miRNA processing, to mRNA splicing, decay, and translation 9, 17. As a RNA helicase, DDX5 acts as a pro-viral host factor in biosynthesis of several RNA viruses, including HIV and HCV 52. By contrast, DDX5 has antiviral function in myxoma virus biosynthesis 53 and HBV biosynthesis 7. In our earlier studies, we have observed that DDX5 protein levels decrease in HBV replicating/infected hepatocytes 7. Moreover, DDX5 knockdown in HBV infected HepG2-NTCP cells increased viral transcription, while the level of H3K27me3, a transcriptionally silencing histone modification associated with cccDNA, was reduced 7. Since DDX5 interacts with the silencing PRC2 complex 7, we interpret these results to mean that DDX5 is involved in epigenetically regulating chromatin modifications of the viral minichromosome. Further studies are needed to determine the cellular context of this regulation.

Transcriptomic and functional analyses reveal that downregulation of DDX5 in HepAD38 hepatocytes results in activation of Wnt/β-catenin signaling **(Figures 4-7)**, a pathway involved in reprogramming of hepatocytes towards a hCSC phenotype in HCCs 18, 44, 54. In addition, the Wnt/β-catenin pathway is an important regulator of adult liver size, liver regeneration, and metabolic zonation 55, 56. Whether DDX5 has a role in regulation of normal liver size, regeneration, and liver zonation is presently unknown. Importantly, recent studies have demonstrated the role of epigenetic mechanisms involving the PRC2 complex in liver regeneration 57.

In this study, we show that downregulation of DDX5 during the course of HBV infection is mediated by miR106b~25 22 and miR17~92 21 **(Figure 1)**. These miRNAs are upregulated during HBV replication** (Figure 2)** and in HBV-related HCCs 23. However, their role in HBV replication and virus-mediated hepatocarcinogenesis has been unknown. These miRNAs suppress pro-apoptotic functions of TGF-β pathway 31, downregulate tumor suppressors PTEN 22 and LKB1 34, and have important roles in maintenance of pluripotency, progenitor cell growth, and regulation of normal development 58. Interestingly, as we demonstrate herein, HBV-induced miR106b~25 and miR17~92 target the same seed sequence in 3'UTR of *DDX5* and downregulate DDX5 during HBV replication** (Figure 1)**. The increased expression of *MCM7* encoding miR106b~25 22 and c-Myc-driven transcription of miR17~92 21, occurring during HBV replication, offer mechanistic insights into the observed induction of these miRNAs** (Figure 2)**. Regarding the question of how HBV replication increases expression of these two miRNA clusters, it is well established that the viral HBx protein functions as an activator of cellular mitogenic pathways 32, 33, 59.

HBV-related HCCs display upregulated expression of these miRNAs 23. The highest induction of miR106b~25 and miR17~92 is observed in HBV-related HCCs that display the lowest *DDX5* mRNA level** (Figure 2D)**. Importantly, HBV-related HCCs with low* DDX5* mRNA belong to “Group III” tumors that express the hCSC-like gene signature, associated with poor patient prognosis 14. The expression levels of miR106b~25, miR17~92 and *DDX5* in these two groups of tumors, namely, “Group III” vs. the “Rest”** (Figure S2C)**, display statistically significant differences **(Figure 2D)**, suggesting a link of DDX5 loss to a hCSC-like phenotype. Mechanistically, loss of function studies of DDX5 demonstrate that DDX5 is multifunctional, having a role in viral biosynthesis **(Figure 3A)**, in drug resistance, and hepatocyte stemness** (Figure 3B and C)**. Specifically, HBV replicating DDX5^KD^ cells form hepatospheres when grown in low attachment plates, are resistant to cisplatin and sorafenib, and express elevated levels of pluripotency genes and hCSC marker CD44** (Figure S4C)**. Moreover, loss of DDX5 either by overexpression of miR106b~25 and miR17~92, stable knockdown of DDX5, or DDX5 siRNA transfection, activate Wnt/β-catenin signaling in various liver cancer cell lines** (Figure 5)**.

The transcriptomic studies comparing the mRNA expression profile of DDX5^KD^ cells, as a function of HBV replication and sorafenib treatment, further support the role of DDX5 as an upstream regulator of Wnt pathway activation. The RNA-seq analysis identified deregulated expression of several *FZD* receptors, as well as regulators of Wnt signaling, upon DDX5 knockdown **(Figures 6 and 7)**. The deregulated expression of Wnt pathway genes including *FZD7, DVL1, SFRP4,* and *SFRP5,* found in our study, agrees with similarly deregulated expression of *FZD7* and* SFRP5* observed in HBV-related HCCs 60, and the upregulated expression of *DVL1* in all HCC types 51. Significantly, upregulated expression of *DVL1*, a key activator of Wnt signaling 49, 50, correlates with reduced *DDX5* expression in HCCs and HCC-derived LIMORE cell lines **(Figure 7)**. Furthermore, downregulation of DDX5 alters chromatin accessibility, enhancing chromatin accessibility near genes of the Wnt pathway** (Figure 4)**. Thus, based on the well-established role of Wnt pathway activation in cellular reprogramming and pluripotency 41, our studies provide a mechanistic link of DDX5 loss to hepatocyte reprogramming/stemness in HCC.

The mechanism by which DDX5 effects chromatin changes likely involves binding to noncoding RNAs 9, and interaction with epigenetic complexes including PRC2 7. Further studies are required to address this mechanism and the role of DDX5 in regulating HBV biosynthesis. DDX5 could regulate viral transcription from cccDNA 7 and/or translation of viral transcripts. Similarly, how DDX5 regulates stemness is incompletely understood. DDX5 was shown to act as a roadblock of somatic cell reprogramming by processing miR-125b, which in turn represses RING1 and YY1 Binding Protein (RYBP), a known inducer of pluripotency-associated genes 39. We also reported earlier that DDX5 is a positive regulator of PRC2 stability 7, and loss of PRC2 function during HBV replication leads to activation of Wnt signaling and re-expression of a hCSC-like gene signature 14. Hence, restoring DDX5 expression in chronically infected hepatocytes could suppress re-expression of the hCSC-like phenotype, providing therapeutic benefit. Considering this important role of DDX5, antagomirs against miR106b~25 and miR17~92 restore DDX5 levels. Significantly, these antagomirs exert both antiviral effects reducing expression of HBV pgRNA and HBc, as well as anti-tumor effects restoring tumor suppressor PTEN and LKB1 **(Figure 8)**. Additionally, antagomir-mediated rescue of *DDX5* reversed Wnt/β-catenin activation in HBV replicating cells, determined by loss of nuclear localization of β-catenin and reduction in *DVL1* expression **(Figure 8)**.

## Conclusion

The results reported herein identify DDX5 as a promising therapeutic target, and also suggest that antagomirs against miR106b~25 and miR17~92 can be explored as therapeutic strategy to suppress DDX5 downregulation. This strategy of therapeutic antagomirs to restore DDX5 levels in HBV infected hepatocytes targets multiple pathways important for HCC, namely, (i) inhibition of HBV replication/biosynthesis (ii) rescue of tumor suppressor genes, and (iii) repression of Wnt signaling. Several miRNA-targeted therapeutic delivery strategies have reached clinical development 61, including lipid-based nanoparticle formulations 62. Hepatocyte-specific deliveries include miRNA-conjugation to cholesterol 63, 64, and N-acetyl-glucosamine (GalNac) which binds with high affinity to the asialoglycoprotein receptor expressed in hepatocytes 61, 65. Recent studies have also developed folate-linked miRNAs targeting folate receptor overexpressing cancer cells 66. Ongoing studies are investigating folate receptor expression in HBV infected hepatocytes. Thus, several promising approaches are available to explore antagomir-mediated restoration of DDX5 in chronically infected HBV patients.

## Supplementary Material

Supplementary figures and tables.

Supplementary table S5, S6.

## Abbreviations

DDX5: DEAD-box helicase 5/p68
HBV: hepatitis B virus
HCC: hepatocellular carcinoma
HBc: hepatitis B virus core antigen
DVL1: Disheveled 1
miR: microRNA
ATAC-seq: assay for transposase-accessible chromatin sequencing
GSEA: gene set enrichment analysis
FDR: false discovery rate
qRT-PCR: quantitative real-time polymerase chain reaction
ChIP: Chromatin immunoprecipitation
TCGA: the cancer genome atlas
LIMORE: liver cancer model repository
PRC2: polycomb repressive complex 2
EpCAM: epithelial cell adhesion molecule
MCM7: minichromosome maintenance complex
PTEN: phosphatase and tensin homologue
LKB1: serine/threonine protein kinase 11
FZD7: frizzled class receptor 7
SFRP: secreted frizzled-related protein
TCF7L1: transcription factor 7-like1
