# Supplementary material for: Restoration of RNA helicase DDX5 suppresses hepatitis B virus (HBV) biosynthesis and Wnt signaling in HBV-related hepatocellular carcinoma
Source: Theranostics. 2020 Sep 1;10(24):10957–72. doi: 10.7150/thno.49629 (PMC7532671; doi:10.7150/thno.49629)
Supplement: Supplementary file 1 — Supplementary figures and tables. [file thnov10p10957s1.pdf]

Supplementary Material

"Restoration of RNA helicase DDX5 suppresses hepatitis B virus (HBV) biosynthesis and Wnt signaling in HBV-related hepatocellular carcinoma"

Saravana Kumar Kailasam Mani[1, 4*], Bingyu Yan [2, 4*], Zhibin Cui[1, 4], Jiazeng Sun[1, 4], Sagar Utturkar[4], Adrien Foca[5], Nadim Fares[6], David Durantel[5], Nadia Lanman[4], Philippe Merle[6], Majid Kazemian[2, 3#], and Ourania Andrisani[1, 4#]

[1]Department of Basic Medical Sciences, [2]Department of Biochemistry, [3]Department of computer Science and [4]Purdue Center for Cancer Research, Purdue University, West Lafayette, IN 47907, [5]Cancer Research Center of Lyon UMR Inserm 1052 - CNRS 5286, and [6]Department of Hepatology, Hôpital de la Croix-Rousse, Hospices Civils de Lyon, Université Lyon 1, Lyon, France

#corresponding authors: kazemian@purdue.edu, andrisao@purdue.edu

*equal contribution

Supplementary Materials includes:

Supplementary Methods

Supplementary Figures S1-S6

Supplementary Tables S1-S4

Supplementary Tables S5-S6 (excel file)

Supplementary Methods

Immunoblotting: performed as previously described [1]. Cells were lysed (15 min, 4°C) in lysis buffer (Cell Signaling), sonicated on ice for 30 sec and clarified by centrifugation (13,000 rpm, 15 min, 4°C). Protein concentration was determined using BCA assay. All samples were diluted to 1µg/µL using 4x dye (Biorad) and equal amounts of proteins (5 µg-40 µg per lane) were run on SDS-PAGE. Following electrophoresis, proteins were transferred to nitrocellulose membrane via wet transfer (200 mA, 45-90 min at 4°C). Following transfer, membranes were blocked with 3% (w/v) BSA in Tris-buffered saline containing 0.1% (v/v) Tween 20 (TBST) and incubated with primary antibody in 3% (w/v) BSA in TBST for 1hr at room temperature, followed by incubation in secondary antibody (1:2000 dilution) in 3% BSA in TBST for 1h at room temperature. Three washes were performed after primary and secondary antibody incubations. Protein bands were detected by chemiluminescence using Pierce ECL (Biorad). Densitometric analysis of immunoblots was performed using ImageJ. Immunoblots are representative of three independent experiments. Antibodies used are listed in Supplementary Table S2:

Immunofluorescence microscopy: was performed as described [1]. Cells ($1x10^5$) were seeded on coverslips in 12-well plates and incubated at 37°C overnight. miRNA inhibitors were transfected using RNAimax and Lipofectamine 3000 on day 3. On day 5, cells were fixed using 4% PFA for 10 min at room temperature. Following fixation, cells were permeabilized with Phosphate Buffered Saline (PBS) containing 0.1% (v/v) Triton X-100 for 15 min at room temperature. Blocking was done using 10% goat serum in PBS containing 0.1% (v/v) Tween-20 (PBST) for 1 h at room temperature. Primary antibodies diluted in blocking solution were used to detect HBc or beta-catenin for 1 h at room temperature. Secondary antibodies conjugated to fluorophores were

diluted in blocking solution and incubated for 45 min at room temperature. Three washes (5 min each) were performed after primary and secondary antibody incubations. A 1 min incubation with Hoechst diluted in PBST to a final concentration of 2 μg/mL to stain nuclei. Coverslips with cells were inverted and mounted in Antifade (10μl) on a glass slide. Microscopy was performed using an Olympus Fluoview confocal microscope. Antibodies used are listed in supplementary Table S2.

RNA preparation and qRT-PCR: RNA was isolated using Purelink mRNA Mini kit (Life Technologies) or Direct Zol RNA miniprep kit (Zymo Research). cDNA synthesized from 1.0 μg total RNA using iSCRIPT cDNA synthesis kit (Biorad). qRT-PCR performed using SYBR green (Roche) in triplicates, normalized to GAPDH. For miRNA quantification, RNA was extracted using miRNeasy Mini Kit (Qiagen) or Trizol for liver tissues from HBV patients with HCC (tumor and peritumoral tissue). Liver tumor samples were obtained from the French National Biological Resources Centre following approved consent from the French Liver Tumor Network Scientific Committee. cDNA synthesized from 1.0 μg RNA using HiSpec buffer in miScript II RT Kit (Qiagen), and qRT-PCR performed using miScript SYBR® Green PCR Kit (Qiagen).

ATAC-Seq and data analysis: HepAD38 cells, wild type (WT) and DDX5 knockdown (KD5), were grown for 10 days. Cells (5 x 10^4) were washed in cold PBS and lysed in 50 μl of lysis buffer (10 mM Tris HCl pH 7.5, 10 mM NaCl, 3 mM $MgCl_2$, 0.1% IGEPAL CA-630). Following lysis and centrifugation, nuclei were used for transposition using Illumina Tagment DNA TDE1 Enzyme and Buffer Kit (Cat# 20034197) per manufacturer's protocol. The transposed DNA was cleaned using Qiagen Reaction Cleanup kit (Cat# 28204), eluted in 10 μL elution buffer and stored at -20°C. Transposed DNA was PCR amplified using primers containing unique adapters. Amplified library was purified using Qiagen MinElute PCR Purification Kit (Cat#28004). Paired-end 2x50 bp sequencing was performed using a HiSeq2500 system (Illumina). Data quality control

was performed using FastQC v0.11.8. Raw sequencing reads were mapped to hg19 using bowtie2.3.4.3 [2] with “-X 2000”. All other parameters were kept as default. Duplicate and low quality (MAPQ<30) reads are removed using PicardTools2.21.6 [3]. Differential chromatin accessible regions/peaks between DDX5 WT and KD were obtained using MACS2.1.2 [4]. The peaks with significant q-value (q<0.01) were retained. SeqMINER1.3.4 [5] was used to cluster the peaks and generate the heatmap of differential peaks. HOMER4.9.1 [6] was used to annotate the peaks with their adjacent genes that were then subjected to pathway analysis against all biological processes from gene ontology (GO) using Matescape [7]. The IGV tracks were made using bamCoverage from DeepTools3.3.0 [8] with parameter “-p 3 -bs 5 --normalizeUsing CPM --ignoreDuplicates” and visualized by IGV browser [9].

References

1. Mani SK, Zhang H, Diab A, Pascuzzi PE, Lefrancois L, Fares N, Bancel B, et al. EpCAM-regulated intramembrane proteolysis induces a cancer stem cell-like gene signature in hepatitis B virus-infected hepatocytes. J Hepatol 2016;65:888-898.

2. Langmead, Ben, and Steven L. Salzberg. Fast gapped-read alignment with Bowtie 2.Nat Methods 9.4 (2012): 357.

3. “Picard Toolkit.” 2019. Broad Institute, GitHub Repository. http://broadinstitute.github.io/picard/; Broad Institute

4. Zhang, Yong, et al. Model-based analysis of ChIP-Seq (MACS). Genome Biol. 9.9 (2008): R137.

5. Ye, Tao, et al. seqMINER: an integrated ChIP-seq data interpretation platform. Nucleic Acids Res. 2011, 39.6; e35-e35.

6. Heinz S, Benner C, Spann N, Bertolino E et al. Simple Combinations of Lineage-Determining Transcription Factors Prime cis-Regulatory Elements Required for Macrophage and B Cell Identities. Mol Cell 2010, 38, 576-589.

7. Zhou, Y., Zhou, B., Pache, L. *et al.* Metascape provides a biologist-oriented resource for the analysis of systems-level datasets. Nat Commun. 2019, 10**,** 1523

6. Ramírez, Fidel, et al. deepTools: a flexible platform for exploring deep-sequencing data. Nucleic Acids Res. 2014, 42.W1: W187-W191.

7. Robinson, James T., et al. Integrative genomics viewer. Nature Biotech. 2011, 29.1: 24.

Supplementary Figures

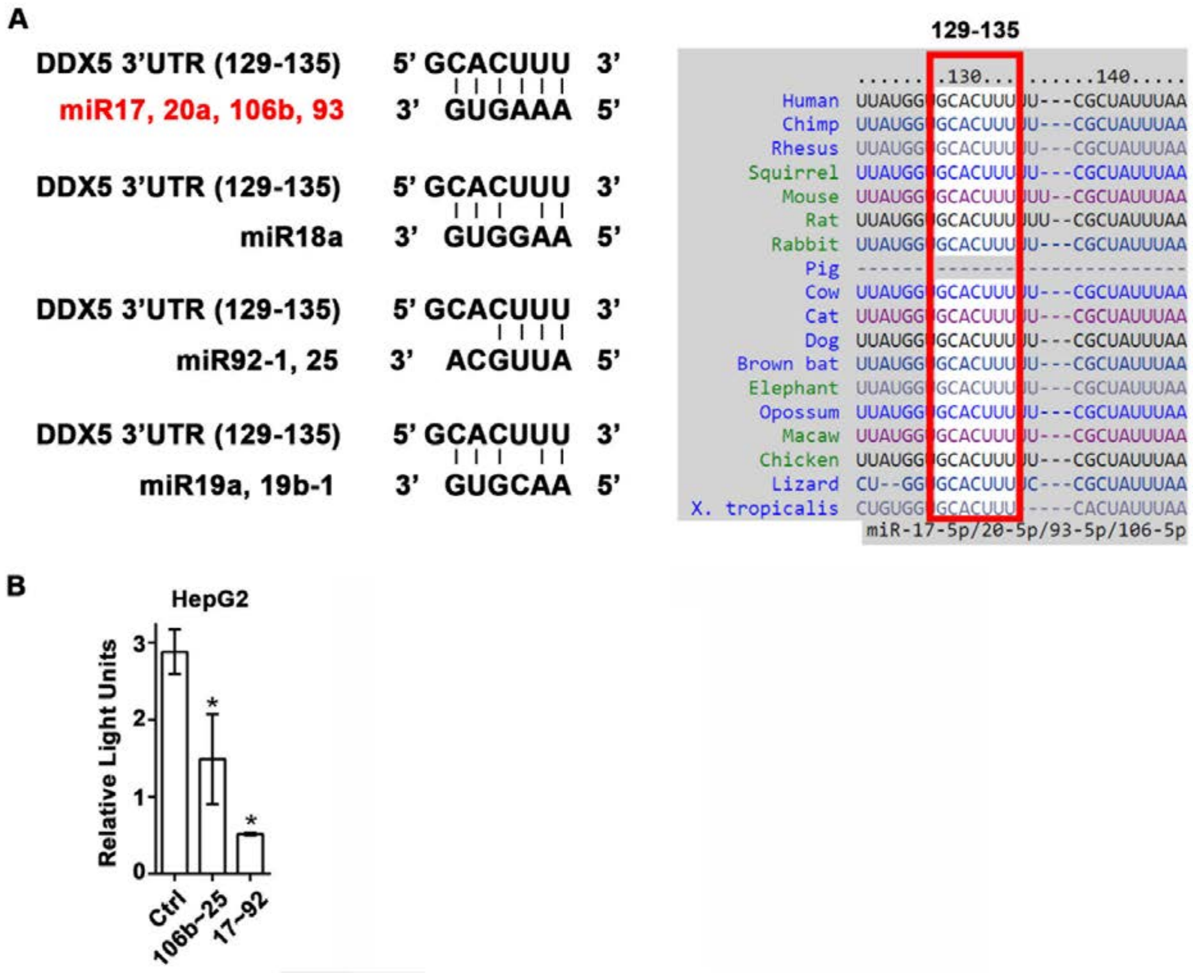

Figure S1 (A) (Left panel) Conserved seed sequences of miR17~92 and miR106b~25 in 3'UTR of DDX5, across 18 species. (Right panel), 3'UTR of DDX5 illustrating the complementarity of the seed sequence for each of the indicated miRNAs. (**B)** Transient transfections of Luc-3'UTR-DDX5 co-transfected with Renilla luciferase, and plasmids expressing mir106b~25 or miR-17~92 in HepG2 cells.

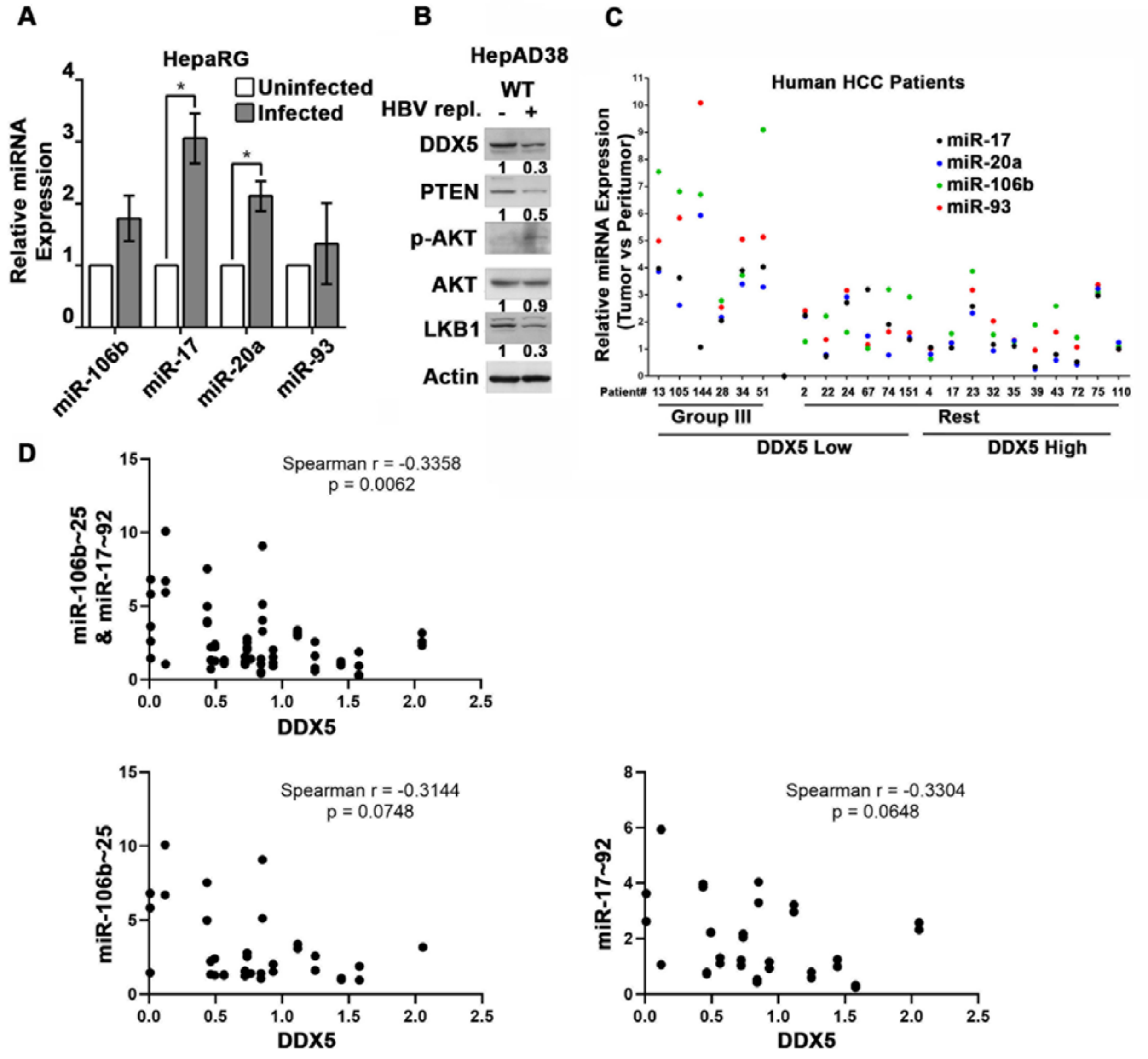

Figure S2 (A) qRT-PCR of indicated miRNAs in HBV-infected HepaRG cells on day4 post-infection (p.i.) (n=3 independent biological replicates). (**B**) Immunoblots of indicated proteins without (-) or with (+) HBV replication for 5 days, in WT HepAD38 cells.(**C**) RT PCR quantification of indicated miRNAs in human HBV-related HCC samples in comparison to peritumor. Tumor samples are classified into Group III *vs.* Rest [1], and DDX5 ^{high} *vs*. DDX5 ^{low} [2]. * $p<0.05$. **(D)** Scatter plots show Spearman correlation between indicated miRNAs and DDX5 for HBV-related HCCs samples from Figs. 2D and S2B.

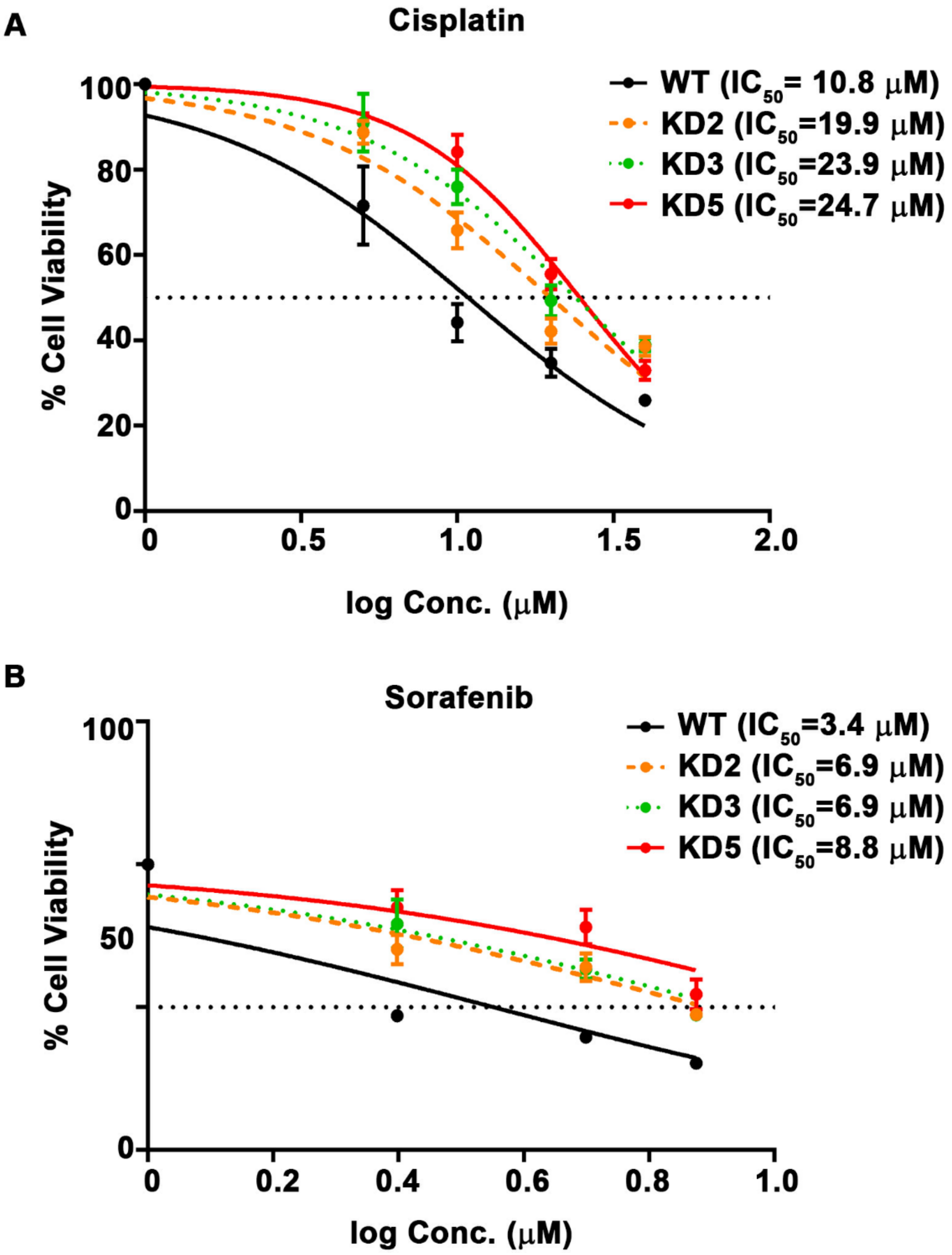

Figure S3: Dose response curves for cisplatin and sorafenib using HepAD38 cells, treated with the indicated concentrations for 2 days, n=3.

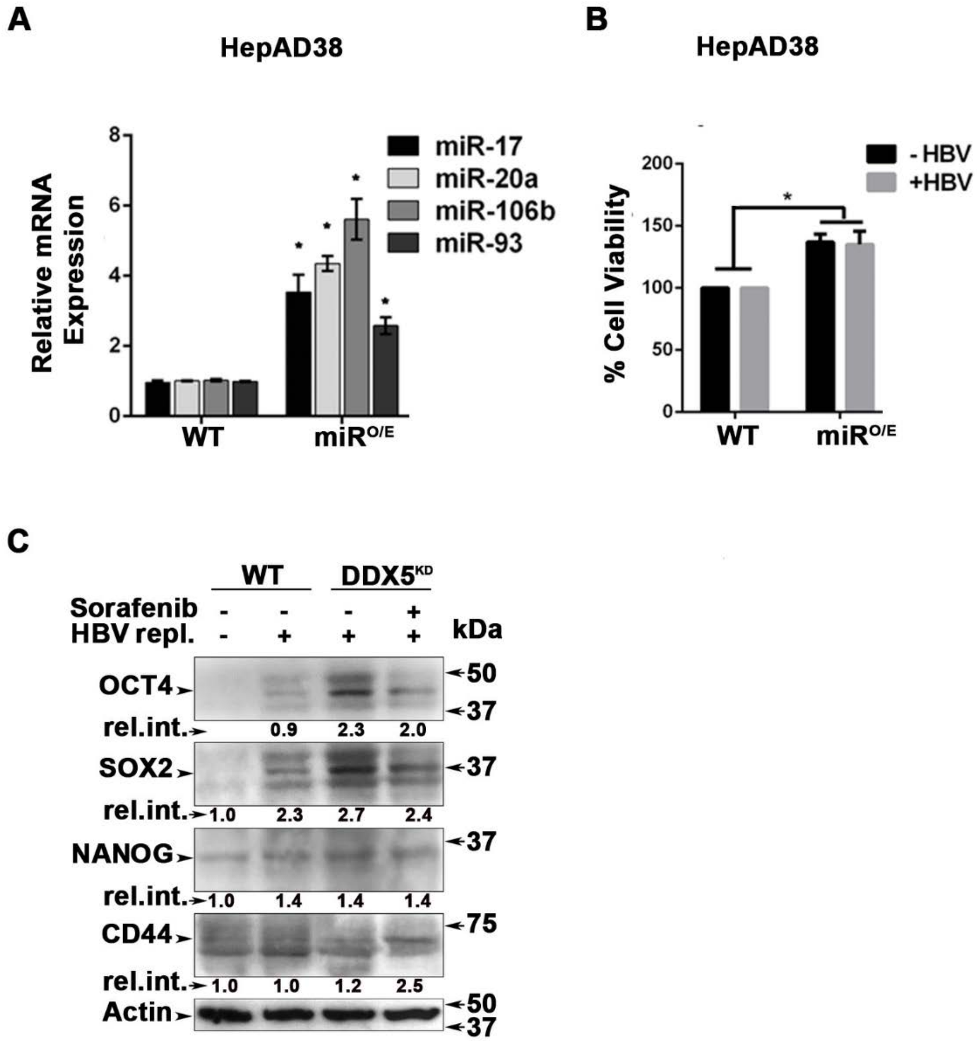

Figure S4: **(A)** Real time PCR analysis of miRNA expression in WT and stable miR17~92 and miR106b~25 overexpressing HepAD38 cells (miR$^{O/E}$). **(B)** MTS assay measuring proliferation of WT and stable miR17~92 and miR106b~25 overexpressing HepAD38 cells without or with HBV replication. (**C)** Immunoblots of indicated proteins in WT and DDX5^{KD} HepAD38 hepatospheres grown with (+) HBV replication and sorafenib treatment, as indicated.

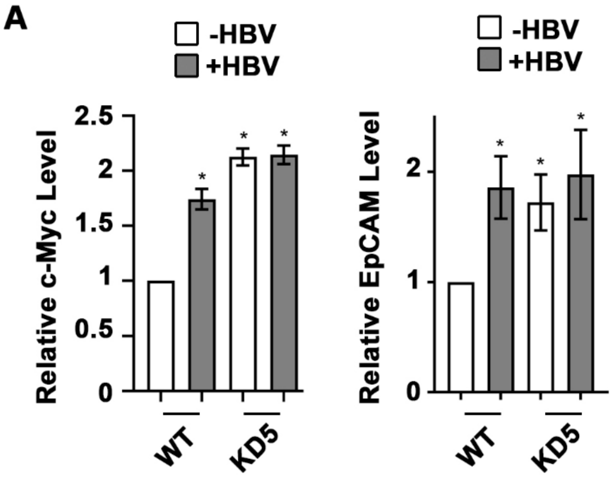

Figure S5: Quantification immunoblots by ImageJ software of indicated proteins in WT and DDX5^{KD} cells with (+) and without (-) HBV replication for 5 days. Results are from three biological replicates.

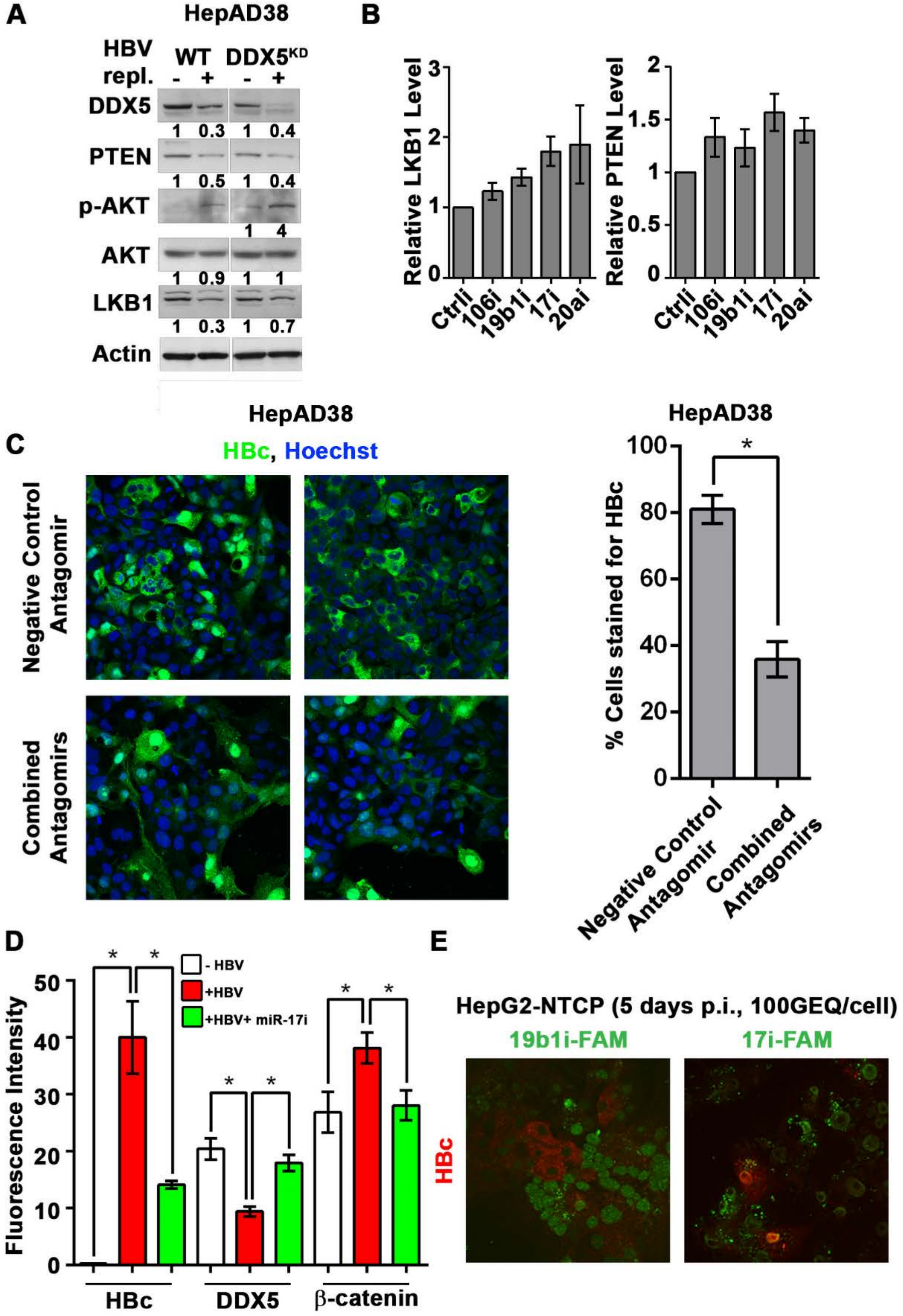

A
HepAD38
HBV repl.
WT
DDX5KD
DDX5
PTEN
p-AKT
AKT
LKB1
Actin
B
Relative LKB1 Level
Relative PTEN Level
Ctrli
106i
19b1i
17i
20ai
C
HepAD38
HBc, Hoechst
Negative Control Antagomir
Combined Antagomirs
% Cells stained for HBc
D
Fluorescence Intensity
- HBV
+HBV
+HBV+ miR-17i
HBc
DDX5
β-catenin
E
HepG2-NTCP (5 days p.i., 100GEQ/cell)
19b1i-FAM
17i-FAM
HBc

Figure S6: **(A)** Immunoblots of indicated proteins without (-) or with (+) HBV replication for 5 days, in WT and $DDX5^{KD}$ HepAD38 cells. **(B)** Quantification of immunoblot data from three independent biological replicates for the indicated proteins from Fig. 8A. **(C)** Immunofluorescence staining of HBc in HepAD38 cells with HBV replication for 5 days. Negative control inhibitor or inhibitor cocktail containing miRNA inhibitors against miR-106b, miR-17, miR-20a and miR-19b1 was transfected on day 3 of HBV replication. (Right Panel) Quantification of percentage of cells showing HBc staining from 3 independent biological replicates. *$p<0.05$; Error bars indicate Mean ± SEM. **(D)** Quantification by ImageJ software of fluorescence intensities of HepAD38 cells transfected with fluorescent antagomir miR-17i-FAM (50 nM). Antagomir transfected on day4 of HBV replication; cells were fixed and immunostained on day 5 p.i. *$p<0.05$; Error bars indicate Mean ± SEM. **(E)** Immunofluorescence staining of HBc in HepG2-NTCP cells infected with 100 genome equivalents of HBV per cell. FAM conjugated miRNA inhibitor against miR-19b1 or miR-17 (green) was transfected on day 6 post infection.

Supplementary Table S1: List of Plasmids and siRNAs

Plasmids, siRNAs	Source
TOPFlash vector [1]	Addgene (12456)
FOPFlash vector [1]	Addgene (12457)
Renilla luciferase vector [2]	Addgene (27163)
Empty vector [3]	Dr. H. Ford: Oncogene. 31, 5162-5171.
miR106b~25 vector [3]	Dr. H. Ford: Oncogene. 31, 5162-5171.
miR17~92 vector [4]	Addgene (21109)
Firefly luciferase - 3'UTR DDX5 in pMirTarget vector	Origene (#SC215943)
Ctrlsi	ThermoFisher Scientific (#4390843)
DDX5si#1	ThermoFisher Scientific (#4392420, assay id s4007)
DDX5si#2	ThermoFisher Scientific (#4392420, assay id s4008)
hsa-miR-19b-1-5p miRCURY LNA miRNA Power Inhibitor	Qiagen (Product# 339131, Catalog# YI04100832-DDB)
hsa-miR-17-5p miRCURY LNA miRNA Power Inhibitor	Qiagen (Product#339131; Catalog# YI04100215-DDB)

1. Veeman MT, Slusarski DC, Kaykas A, Louie SH, Moon RT. Zebrafish prickle, a modulator of noncanonical Wnt/Fz signaling, regulates gastrulation movements. Curr Biol.2003, 13(8):680-5.
2. Chen X, Prywes R. Serum-induced expression of the cdc25A gene by relief of E2F-mediated repression. Mol Cell Biol. 1999, 19(7):4695-702.
3. Smith, A.L., Iwanaga, R., Drasin, D.J., Micalizzi, D.S., Vartuli, R.L., Tan, A.C., and Ford, H.L. The miR-106b-25 cluster targets Smad7, activates TGF-beta signaling, and induces EMT and tumor initiating cell characteristics downstream of Six1 in human breast cancer. Oncogene. 2012, 31, 5162-5171.
4. O'Donnell KA, Wentzel EA, Zeller KI, Dang CV, Mendell JT. c-Myc-regulated microRNAs modulate E2F1 expression. Nature.2005, 435(7043):839-43.

Supplementary Table S2: Antibodies Used

Antibody	Dilution	Application	Source
Rabbit α-Human DDX5	1:1000 in 3% BSA in TBST	Western Blot	Cell Signaling Technologies (#9877S)
Mouse α-Human Actin	1:2000 in 3% BSA in TBST	Western Blot	Sigma (#A5441)
Rabbit α-Human MCM7	1:1000 in 3% BSA in TBST	Western Blot	Cell Signaling Technologies (#3735S)
Rabbit α-Human OCT4	1:1000 in 3% BSA in TBST	Western Blot	Cell Signaling Technologies (#2750S), Abcam (#ab19857)
Mouse α-Human SOX2	1:1000 in 3% BSA in TBST	Western Blot	Cell Signaling Technologies (#4900S)
Rabbit α-Human NANOG	1:1000 in 3% BSA in TBST	Western Blot	Cell Signaling Technologies (#4903S)
Mouse α-Human CD44	1:1000 in 3% BSA in TBST	Western Blot	Cell Signaling Technologies (#3570S)
Rabbit α-Human PTEN	1:1000 in 3% BSA in TBST	Western Blot	Cell Signaling Technologies (#9559S), R&D Systems (# AF847)
Rabbit α-Human LKB1	1:1000 in 3% BSA in TBST	Western Blot	Cell Signaling Technologies (#3047S), Novus Biologicals (#NBP2-14835SS)
Rabbit α-Human Akt	1:1000 in 3% BSA in TBST	Western Blot	Cell Signaling Technologies (#4691S)
Rabbit α-Human p-Akt	1:1000 in 3% BSA in TBST	Western Blot	Cell Signaling Technologies (#4060S)
Mouse α-Human SMAD7	1:1000 in 3% BSA in TBST	Western Blot	R&D Systems (# MAB2029)
Rabbit α-HBV Core	1:5000 in 3% BSA in TBST	Western Blot	Dr. Adam Zlotnick

Horse α-Mouse secondary	1:2000 in 3% BSA in TBST	Western Blot	Vector Laboratories (#PI-2000)
Goat α-Rabbit secondary	1:2000 in 3% BSA in TBST	Western Blot	Vector Laboratories (#PI-1000)
Rabbit IgG	5μg	ChIP	Cell Signaling Technologies (#2729S)
Rabbit α-Human c-Myc	5μg	ChIP	Cell Signaling Technologies (#9402S)
Rabbit α-Human DDX5	1:100 in 10% Goat serum in PBST	IF	Cell Signaling Technologies (#9877S)
Mouse α-Human β-catenin	1:100 in 10% Goat serum in PBST	IF	Cell Signaling Technologies (#2677S)
Rabbit α-HBV Core	1:1000 in 10% Goat serum in PBST	IF	Dr. Adam Zlotnick
Goat α-Rabbit Alexa Fluor 633	1:2000 in 10% Goat serum in PBST	IF	ThermoFisher Scientific (#A21070)
Goat α-Rabbit Alexa Fluor 594	1:2000 in 10% Goat serum in PBST	IF	ThermoFisher Scientific (#A11012)
Goat α-Rabbit Alexa Fluor 488	1:2000 in 10% Goat serum in PBST	IF	ThermoFisher Scientific (#A11008)
Goat α-Mouse Alexa Fluor 633	1:2000 in 10% Goat serum in PBST	IF	ThermoFisher Scientific (#A21050)

Supplementary Table S3: Primer sequences

Primer	5’ - Sequence - 3’
DDX5 Forward	AGCAAGTGAGCGACCTTATC
DDX5 Reverse	CATCCTTCATGCCTCCTCTAC
EpCAM Forward	TCGTCAATGCCAGTGTACTTC
EpCAM Reverse	GCCATTCATTTCTGCCTTCATC
AFP Forward	AGACTGAAAACCCTCTTGAATGC
AFP Reverse	GTCCTCACTGAGTTGGCAACA
OCT4 Forward	GTG TTC AGC CAA AAG ACC ATC T
OCT4 Reverse	GGC CTG CAT GAG GGT TTC T
SOX2 Forward	TGG ACA GTT ACG CGC ACA T
SOX2 Reverse	CGA GTA GGA CAT GCT GTA GGT
NANOG Forward	TTT GTG GGC CTG AAG AAA ACT
NANOG Reverse	AGG GCT GTC CTG AAT AAG CAG
FZD7 Forward	GCCTGATGTACTTTAAGGAGGAG
FZD7 Reverse	CAGGTAGGTGAGAACGGTAAAG
WNT7B Forward	TCTACGTGTTTCTCTGCTTTGG
WNT7B Reverse	GCTAGGCCAGGAATCTTGTT
SFRP4 Forward	GCCAACTTTGGCAACGTATC
SFRP4 Reverse	CCACCGTTGTGACCTCATT
SFRP5 Forward	GATGTGCTCCAGTGACTTTGT
SFRP5 Reverse	GGCTTGAGCAGCTTCTTCTT
DVL1 Forward	GACTCATCCGGAAGCACAAA
DVL1 Reverse	GACATGGTGGAGTCGGTTATG

DVL3 Forward	CGGCATCTACATTGGCTCTATC
DVL3 Reverse	CGGACTGCATCGTCATTACTC
MMP7 Forward	GCTCACTTCGATGAGGATGAA
MMP7 Reverse	AGGAATGTCCCATACCCAAAG
GAPDH Forward	CCCTTCATTGACCTCAACTACA
GAPDH Reverse	ATGACAAGCTTCCCGTTCTC
HBc Forward	2112F 5'-CTGGGTGGGTGTTAATTTGG
HBc Reverse	2317R 5'-TAGGGGCATTTGGTGGTCTA
HBV pgRNA Forward	383F 5'-CTCCTCCAGCTTATAGACC<br>Xia et al, 2019 **PMID:30021897**<br>Lucifora et al, 2014 **PMID: 24557838**
HBV pgRNA Reverse	705R 5'-GTGAGTGGGCCTACAAA
Total HBV RNA Forward	1805F 5'-TCACCAGCACCATGCAAC<br>Yan et al; 2012 **PMID: 23150796**
Total HBV RNA Reverse	1896R 5'-AAGCCACCCAAGGCACAG
HBV pgRNA Forward	2270F 5'-GAGTGTGGATTCGCACTCC<br>Xia et al, 2019 **PMID:30021897**
HBV pgRNA Reverse	2392R 5'-GAGGCGAGGGAGTTCTTCT
miR-17 5p Forward	CAAAGTGCTTACAGTGCAGGTAG
miR-18a 5p Forward	TAAGGTGCATCTAGTGCAGATAG
miR-19a 5p Forward	TGTGCAAATCTATGCAAAACTGA
miR-19b1 5p Forward	TGTGCAAATCCATGCAAAACTGA
miR-20a 5p Forward	TAAAGTGCTTATAGTGCAGGTAG
miR-92a1 5p Forward	TATTGCACTTGTCCCGGCCTGT
miR-106b 5p Forward	TAAAGTGCTGACAGTGCAGAT

miR-93 5p Forward	CAAAGTGCTGTTCGTGCAGGTAG
miR-25 5p Forward	CATTGCACTTGTCTCGGTCTGA
U6 snRNA Forward	CTCGCTTCGGCAGCACATATACT
U6 snRNA Reverse	ACGCTTCACGAATTTGCGTGTC
Universal Reverse	Qiagen Proprietary information
c-myc site 1 Forward	ACCTCGGAAACCCACCAAG
c-myc site 1 Reverse	TCTCCCTGGGACTCGACG
c-myc site 2 Forward	AAAGGCAGGCTCGTCGTTG
c-myc site 2 Reverse	CGGGATAAAGAGTTGTTTCTCCAA
c-myc site 3 Forward	CTCGACTCTTACTCTCACAAATGG
c-myc site 3 Reverse	GCTACTGGTGCAGTTAGGTCC
miR-106b 5p inhibitor	mA/ZEN/mU mCmUmG mCmAmC mUmGmU mCmAmG mCmAmC mUmUmU /3ZEN/
miR-17 5p inhibitor	mC/ZEN/mU mAmCmC mUmGmC mAmCmU mGmUmA mAmGmC mAmCmU mUmU/3ZEN/
miR-19b1 inhibitor	mG/ZEN/mC mUmGmG mAmUmG mCmAmA mAmCmC mUmGmC mAmAmA mAmC/3ZEN/
miR-20a inhibitor	mC/ZEN/mU mAmCmC mUmGmC mAmCmU mAmUmA mAmGmC mAmCmU mUmU/3ZEN/
Negative Control inhibitor	mG/ZEN/mC mGmAmC mUmAmU mAmCmG mCmGmC mAmAmU mAmUmG mG/3ZEN/

Supplementary Table S4: Reagents, Chemical inhibitors, and Kits

Reagents, Kits	Source
Cisplatin	Selleck Chemicals (#S1166)
Sorafenib	Selleck Chemicals (#S7397)
XAV-939	Selleck Chemicals (#S1180)
ICG-001	Selleck Chemicals (#S2662)
Dual-Luciferase® Reporter Assay System	Promega (#E1980)
CellTiter 96® AQueous One Solution Cell Proliferation Assay (MTS)	Promega (#G3580)
Cell Lysis Buffer (10X)	Cell Signaling Technology (#9803)
miScript SYBR® Green PCR Kit	Qiagen (#218073)
miScript II RT Kit	Qiagen (#218161)
miRNeasy Mini Kit	Qiagen (#217004)
LightCycler® 480 SYBR Green I Master	Roche (#04887352001)
LightCycler® 8-Tube Strips (white)	Roche (#06612601001)
LightCycler® 480 Sealing Foil	Roche (#04729757001)
4x Laemmli Sample Buffer	Biorad (#1610747)
iScript™ cDNA Synthesis Kit	Biorad (#1708891)
Nitrocellulose Membrane, Roll, 0.2 µm	Biorad (#1620112)
DMSO	Sigma (#D8418-50ML)
Tetracycline hydrochloride	Sigma (#T7660-5G)
Bovine Serum Albumin	Sigma (#A9647-100G)
Triton™ X-100	Sigma (#T8787-100ML)
Costar® 6-well Ultra-Low Attachment Plates	Corning (#3471)

Pierce™ ECL Western Blotting Substrate	ThermoFisher Scientific (#32106)
Geneticin™ Selective Antibiotic (G418 Sulfate)	ThermoFisher Scientific (#10131027)
PureLink™ RNA Mini Kit	ThermoFisher Scientific (#12183025)
Pierce™ BCA Protein Assay Kit	ThermoFisher Scientific (#23227)
Lipofectamine™ 3000 Transfection Reagent	ThermoFisher Scientific (#L3000015)
Lipofectamine™ RNAiMAX Transfection Reagent	ThermoFisher Scientific (#13778150)
Hoechst 33342 Solution (20 mM)	ThermoFisher Scientific (#62249)
Tween™ 20	ThermoFisher Scientific (BP337-500)
ProLong™ Diamond Antifade Mountant	ThermoFisher Scientific (#P36970)
B-27™ Supplement (50X), minus vitamin A	ThermoFisher Scientific (#12587010)
Recombinant Human EGF	Peprotech (#AF-100-15)
Recombinant Human FGF-basic	Peprotech (#100-18C)
Tagment DNA TDE1 Enzyme and Buffer Kit	Illumina (Cat# 20034197)
Reaction Cleanup kit	Qiagen (Cat# 28204),
MinElute PCR Purification Kit	Qiagen (Cat#28004).
